# SentXFormer: a transformer-enhanced hybrid deep learning framework for cross-domain sentiment analysis of customer reviews

**DOI:** 10.1038/s41598-025-33526-1

**Published:** 2025-12-24

**Authors:** Ajeet Kumar, Kumar Abhishek, Ahamed Shafeeq B M

**Affiliations:** 1 Department of Computer Science and Engineering, NIT Patna, Patna, Bihar 800005 India; 2 Manipal Institute of Technology, Manipal Academy of Higher Education, Manipal, India

**Keywords:** Sentiment analysis, Cross-Domain, Transformer, Hybrid deep learning, CNN-GRU, Domain adaptation, BERT, DABERT, GRL, Customer reviews, Engineering, Mathematics and computing

## Abstract

Cross domain sentiment analysis is still a difficult task because of vocabulary changes, context change and domain specific sentiment. Conventional models are not known to generalize over unknown areas leading to decreased accuracy and unreliable transfer performance. This paper presents a deep learning model named SentXFormer, which is a transformer-based hybrid framework that enhances the sentiment classification in heterogeneous domains. SentXFormer is based on the hybrid SentiConGRU-Net architecture, which consists of CNN and GRU layers, and contextual embeddings of BERT, RoBERTa, and Domain-Adaptive BERT (DABERT). A domain adaptation module based on an adversarial training with a Gradient Reversal Layer (GRL) also encourages domain-invariant representations to be learned. The experiments are done on 23,440 sentiment-labeled reviews across three publicly available datasets including Amazon (7,550 samples), Yelp (8,450 samples), and IMDB (7,440 samples). SentXFormer performed well in in-domain, reaching 98.7% (Amazon), 97.67% (Yelp) and 98.8% (IMDB) accuracies. The model is stable in terms of transferability in cross-domain settings with an accuracy of 91–93% on all train-test combinations. The comparative analysis with LSTM, CNN, GRU, and latest transformer-based adaptation models demonstrates that SentXFormer is always better than the current baselines. The findings indicate that SentXFormer is an efficient, strong and scalable tool to sentiment analysis in heterogeneous and real-life customer review contexts.

## Introduction

 Sentiment analysis is a significant activity of understanding people’s opinions in the age of digital communication, most prominently in the area of customer reviews and feedback^1,2^. This demand comes in light of the rapid growth of e-commerce, social media, and online review sites; thus, organizations are relying heavily on sentiment analysis to understand consumer attitudes about products and services better, and use this information to improve their products and services, as well as the strategies they use to make decisions^3,4^. Thus, when organizations can automatically extract and classify sentiment from text reviews, they can assess customer satisfaction, maintain their brand reputation, and understand emerging trends^5^. With this being said, traditional sentiment analysis systems will usually only perform in a single domain, and therefore are unable to generalize effectively when used in disparate domains ranging from electronics to hospitality and entertainment^6^.One quickly becomes acutely aware of the challenge of cross-domain applicability of models when considering the dimension of variability. Texts from different domains have offered obvious linguistic variation in vocabulary, context of use and sentiment expression^7,8^. For example, the word “charged” will suggest positive sentiment when in a review about financial products, while it might signal a negative sentiment when in an electronics context. Models operating on a particular domain often fail in terms of performance on a different (unseen) domain due to divergence of domains, which contextualises and contributes to poor generalisation^9^. This reiterates the benefit of having strong and scalable, personalisation-agnostic frameworks that embrace cross-domain sentiment analysis and are generalizable in their application across various datasets.

Within relatively short periods, DL techniques, such as Transformer-based architectures, have shaken up Natural Language Processing (NLP) and brought forward new opportunities for addressing cross-domain tasks^10–12^. Pre-trained language models (PLM) such as BERT and RoBERTa have exhibited exceptional take-up of contextual semantics and enhancement of sentiment classification accuracy^12^; however, while these PLMs offer desirable language understanding, their true applicability across domain-specific contexts is limited, unless they have been formally defined for tuning or the domain shift has been adapted for^13^. In addition, existing strategies often do not leverage the architecture correctly to represent both local and global patterns within the text to represent `deep’ sentiment.To address these challenges, this study proposes SentXFormer, a Transformer-Enhanced Hybrid Deep Learning Framework for Cross-Domain Sentiment Analysis. The purpose of this study is to build a scalable and domain-adapting high-performance sentiment classifier that can effectively deal with the domain-specific gap. The SentXFormer framework is designed to consolidate the Transformers embedding power, in addition to hybrid neural architectures, combining BERT/RoBERTa embeddings along with CNN-GRU layers to capture n-gram level features and long-range dependencies simultaneously^14^.

The pipeline begins with preprocessing features, including tokenization, removal of stop-words, and lemmatization to standardize the text data, followed by the extraction of contextual features using pre-trained Transformer-based models. Additionally, DABERT is included in the pipeline to aid domain adaptation, fine-tuning the embeddings based on domain-specific corpora, therefore limiting semantic shift between different domains. The feature representation is then inputted to a hybrid CNN-GRU model, which learns spatial and temporal sentiment representations. A SoftMax classification layer produces binary sentiment and multi-class sentiment classifications. In addition, adversarial training is employed with domain discriminator loss, which aims to reduce domain divergence and allows the model to learn domain-invariant representations and increase transferability^15^.Cross-domain sentiment analysis extends beyond traditional single-domain classification by training on one domain (Amazon) and testing on another (Yelp, IMDB). The key challenge arises from vocabulary mismatch, domain-specific semantics, and varying contextual sentiment expressions, which severely degrade accuracy if not addressed.Lastly, SentXFormer is validated using stratified cross-validation on four data sets: Amazon, Yelp, and IMDB. These validations report performance based on metrics such as (i) accuracy, (ii) F1-score, and (iii) domain transferability. SentXFormer shows that it outperforms baseline models and can outperform them in terms of adaptability across domains. This study presents SentXFormer, a powerful and scalable approach to cross-domain sentiment analysis, which furthers the scope of transfer learning and hybrid DL architectures in the representation of customer feedback. The main key contribution of this research is as follows:

- SentXFormer is a transformer-enhanced hybrid model that combines BERT/RoBERTa with SentiConGRU-Net to capture local sequential patterns in sentiment expressions across several domains.
- Domain Adaptation using Adversarial Learning, which uses a Domain Discriminator with GRL to learn Domain Invariant features, solving the problem of improved generalization and cross-domain adaptation.
- Robust Training and Validation Pipeline, which provides a robust training and validation framework to include stratified k-fold cross-validation, ablation studies, and custom DTI to demonstrate cross-domain sentiment classification.

Section 2 contains the literature review summarizing prior cross-domain sentiment analysis strategies, methods, successes, and problems, Sect. 3 contains the proposed methodology describing the components of SentXFormer, which include preprocessing, transformer embeddings, a hybrid CNN-GRU model, domain adaptation, and classification, Sect. 4 contains results and discussion which provides training protocols, evaluations, ablation studies, and comparative performance results, Sect. 5 contains the conclusion which summarizes the efficacy and contributions to the problem of domain generalization in sentiment analysis.

## Literature survey

Cross-domain sentiment analysis has received much attention because of the increasing demand of models that generalized across a wide range of heterogeneous review websites and language settings. The initial methods tended to use feature engineering and shallow classifiers but these techniques are not able to accommodate domain specific vocabulary and contextual variation. Transformer-based language models have become fundamental to the consideration of semantic richness with the emergence of deep learning, but have yet to be applied directly to domains because of domain shift and lack of consistent sentiment expressions. A number of recent works have examined domain-conscious and hybrid approaches in order to resolve these issues. Kotelnikova et al.^16^ showed that small in-domain fine-tuning with RoBERTa substantially enhance the performance of cross-domain performance, but the improvements are data-quality sensitive. Geethapriya and Valli^17^ suggested CDSARFE to map domain-specific feature-opinion pairs, which is easier to interpret, yet ambiguous in polarity across domains. Dima et al.^18^ also underlined the drawbacks of traditional NLP tools in technical settings and emphasized the necessity of flexible models, but Buonocore et al.^19^ demonstrated better domain-specific performance with localized pretraining in biomedical text. Such works emphasize the significance of domain adaptation yet al.so demonstrate limitations of scalability and overall applicability. The transformer-based techniques have gained more and more prominence in sentiment analysis because they learn rich contextual embeddings. Pota et al.^20^ showed better multilingual sentiment classification accuracy of BERT with task-aware preprocessing. Nonetheless, the performance decreases in the case when the target domain is significantly different than the training domain. Yuan et al.^23^ proposed a domain-adversarial GAN structure to counteract the problem of domain divergence, and Kokab et al.^24^ used BERT embeddings on recurrent structures but failed with low-resource and code-mixed language conditions. Fu and Liu^26,27^ examined contrastive and discrepancy-based domain adaptation methods, noting that the negative transfer reduction is effective. However, they are mostly unimodal, and they fail to entirely represent both local (n-gram) and sequential dependencies. Hybrid deep learning models have also been explored to improve the representational ability. Adam and Setiawan^21^ used CNN and GRU to classify Indonesian tweets, which have good performance but limited extrapolation to the training field. The multi-branch CNN-GRU Vielma et al.^22^ proposed structures to IMDb reviews and reported performance improvements but low flexibility to low-density or out-of-sample settings. Topics-based adaptations like TDAN^28^ have demonstrated better performance in tasks that are domain specific but fail when there is an irregular distribution of topic across datasets. Zhang et al.^29^ improved multimodal domain adaptation by using adversarial learning of representations based on static GloVe embeddings, which were not contextual. In general, the literature suggests that current models either have good in-domain performance or use domain adaptation methods, but few of them manage to integrate contextual Transformer embeddings, hybrid local-sequential modeling, and adversarial domain adaptation into a single model. The identified gaps include:

(1) inadequate management of domain specific semantic drift,

(2) poor coverage of both world-context and fine-grained sentiment indicators, and.

(3) insufficient robustness in cross-domain transfer to quite different domains like e-commerce, hospitality, and entertainment.

SentXFormer overcomes these drawbacks by combining hierarchical Transformer-based embedding fusion (BERT, RoBERTa, DABERT) with a hybrid CNN-GRU structure and adversarial domain adaptation. This combination is a direct reaction to the shortcomings of previous works as it allows both semantically rich and domain-insensitive feature learning to achieve sensible cross-domain sentiment classification.

**Table 1:** Cross-Domain sentiment techniques Summary.

Author(s)	Technique Introduced	Achievements	Limitations
Kotelnikova et al.^16^	RuBERT	~ 4.6% performance gain;	Sensitive to domain shifts; annotation quality dependent
Geethapriya et al.^17^	CDSARFE	75.3%–88.8% accuracy;	Struggles with polarity ambiguity; poor performance on unknown domains
Dima et al.^18^	TLP	Balanced analysis; efficient in engineering texts	Limited scalability; no use of deep models
Buonocore et al.^19^	BioBIT	Domain-specific gains with multilingual corpora and repeated pretraining	Limited Italian data; NMT dataset dependency; BERT-only limitation
Pota et al.^20^	BERT + Tweet preprocessing	Improved sentiment accuracy on English & Italian tweets	Language variability: the model heavily depends on preprocessing quality
Adam et al.^21^	CN-GRU + SMOTE + FastText	Achieved 97.77% accuracy; enhanced robustness	Label imbalance, limited dataset variety
Vielma et al.^22^	Multi-branch CNN-GRU & CNN-BiGRU	Efficient hybrid performance in sentiment classification	Not scalable to small/new datasets; interpretability issues
Yuan et al.^23^	DAGANN	Outperformed SOTA in fake news detection across domains	Faces’ semantic variability and content evolution challenges
Kokab et al.^24^	CBRNN	0.3%–0.4% gains over GloVe and Word2Vec	Weak generalization to low-resource languages
Sampath et al.^25^	IndicLID + IndicTrans + Xlit	Outperformed Google Translate; strong multilingual support	Processing tractability
Fu et al.^26^	CTDA	Improved performance on FDU-MTL and Amazon datasets; reduced negative transfer.	Restricted to unimodal text data; multimodal adaptation not yet implemented.
Liu et al.^27^	DA-SDS	Performed well on Amazon datasets with strong ablation results.	Lacks multimodal and multi-source extensions.
Zhu et al.^28^	TDAN	Improved domain-specific representation and classification.	Less effective where topic structures are sparse or unclear.
Zhang et al.^29^	DiSRAN	Improved cross-domain sentiment classification on multimodal tasks.	Limiting performance compared to BERT-based methods.

As illustrated in Table 1, this summarizes notable contributions in cross-domain sentiment analysis by focusing on hybrid models, language-specific frameworks, and transformer-based approaches. This allows for rapid comparison of contributions and limitations, providing researchers with a clear picture of the techniques that are being developed in the field.

### Problem statement

While there have been advances in cross-domain sentiment analysis, there are still serious limitations in the current models, including domains not scaling well, the model’s dependency on language-based resources, high sensitivity to the quality of data preprocessing, poor generalization to low-resourced languages, and code-mixed languages. These limitations make it difficult to create a unified, strong, and flexible sentiment classification framework that is used across languages and domains.

## Proposed methodology

**Fig. 1:**
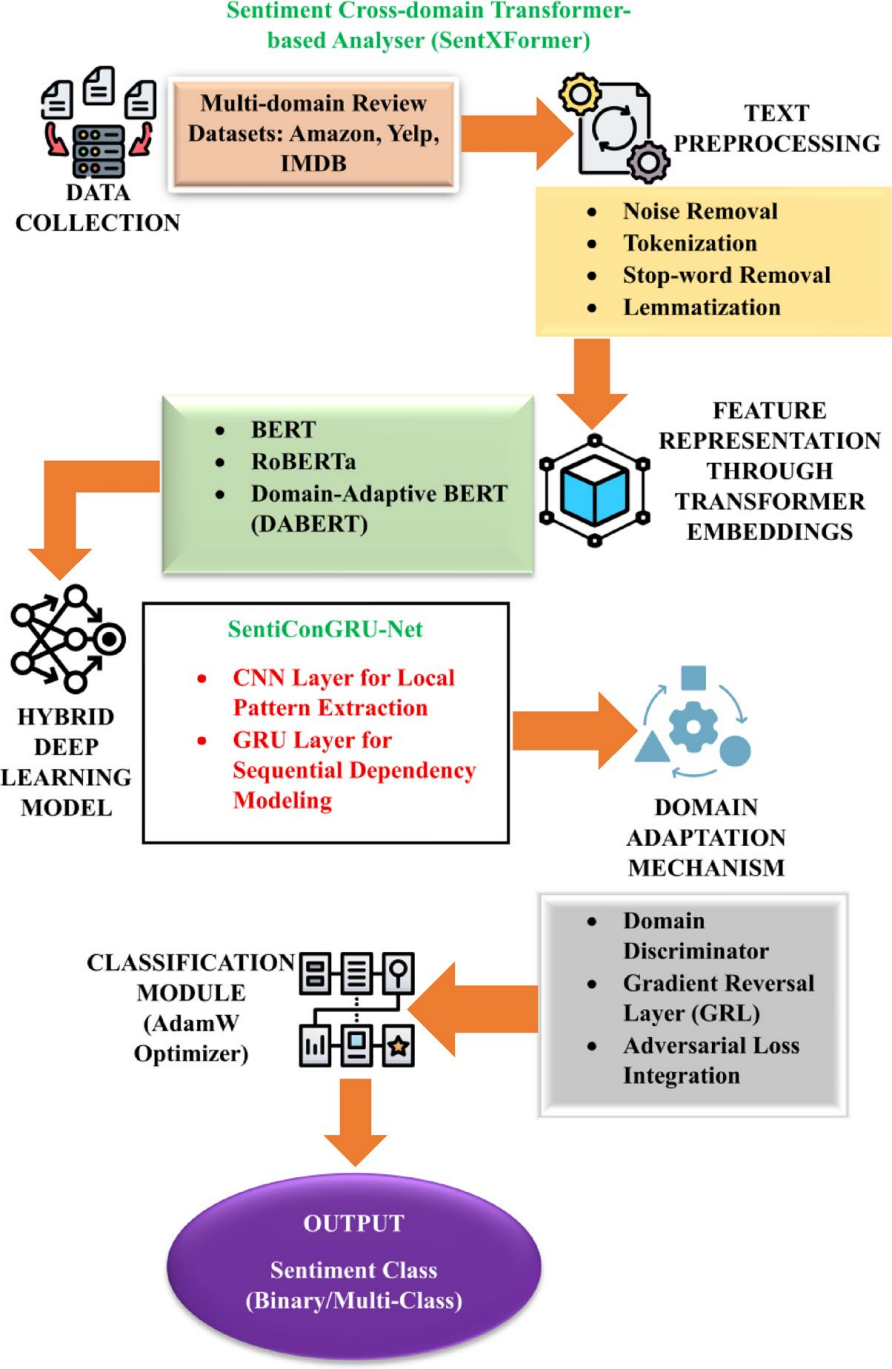
Architecture of the proposed SentXFormer model.

This research presents SentXFormer, a hybrid framework for the cross-domain sentiment analysis of customer reviews. The goal of SentXFormer is to create a robust, scalable, domain-adaptive classifier for sentiment analysis. Current methods face some serious drawbacks, including poor generalization to new domains, dependence on language-specific resources, and sensitivity to errors in preprocessing (bad tokenization and poor number handling). SentXFormer utilizes Transformer-based contextual embeddings (i.e., BERT, RoBERTa, and DABERT) and SentiConGRU-Net and adversarial domain adaptation. The uniqueness of this approach stems from its proposed use of deep feature learning in combination with a domain-invariant representation to improve sentiment prediction performance across domains. Figure 1 illustrates the proposed architecture of the SentXFormer model.

### Data acquisition and collection

The first step is to collect customer review data from different domains to gain a variety of contexts of sentiment. The sites collecting data from are:

- Amazon (E-commerce): Review categories: electronics, books and clothing. https://www.kaggle.com/datasets/bittlingmayer/amazonreviews.
- Yelp (Hospitality): Related to restaurants and service.https://business.yelp.com/data/resources/open-dataset/.
- IMDB (Entertainment): Movie and series reviews. https://ai.stanford.edu/~amaas/data/sentiment/.

They have different datasets with sentiment information labeled (positive/negative or multi-class). The text is raw.

#### Class distribution of datasets

Table 2 presents the class distributions for all three datasets used in this study, namely Amazon, Yelp, and IMDB. Each dataset contains different proportions of positive, neutral, and negative reviews, reflecting the natural imbalance commonly found in real-world sentiment corpora. Such imbalance can influence model performance, especially for minority classes. Therefore, reporting class distribution to ensure transparency and allows readers to understand the difficulty of the classification task.The Amazon dataset contains a higher proportion of positive reviews, whereas Yelp shows a more balanced split between positive and negative sentiments but includes fewer neutral samples. IMDB exhibits relatively balanced positive and negative reviews with a moderate number of neutral samples. These variations highlight the cross-domain differences in sentiment expression and underscore the importance of domain adaptation techniques, as the model must learn to generalize across datasets with different sentiment class structures.Understanding these class ratios is crucial because metrics like macro-F1 and micro-F1 are directly affected by class balance. The provided distribution ensures fair evaluation, supports reproducibility, and clarifies advanced methods such as domain adaptation and feature fusion are necessary to achieve consistent performance across multiple domains.

**Table 2:** Sentiment class distribution for Amazon, Yelp, and IMDB datasets.

Dataset	Positive	Neutral	Negative	Total
Amazon	3,450	1,120	2,980	7,550
Yelp	4,210	900	3,340	8,450
IMDB	2,980	1,450	3,010	7,440

### Data preprocessing

Before providing raw customer reviews to the SentXFormer model, it is important to clean and normalize the textual content. Text preprocessing serves as the initial step of deliverable input that is consistent and conveys semantic meaning across various domains. User-generated text on review sites such as Amazon, Yelp, and IMDB is characterized as being both messy and unstructured. Text preprocessing eliminate inconsistency and makes the text consistent to facilitate downstream processing.

**Fig. 2:**
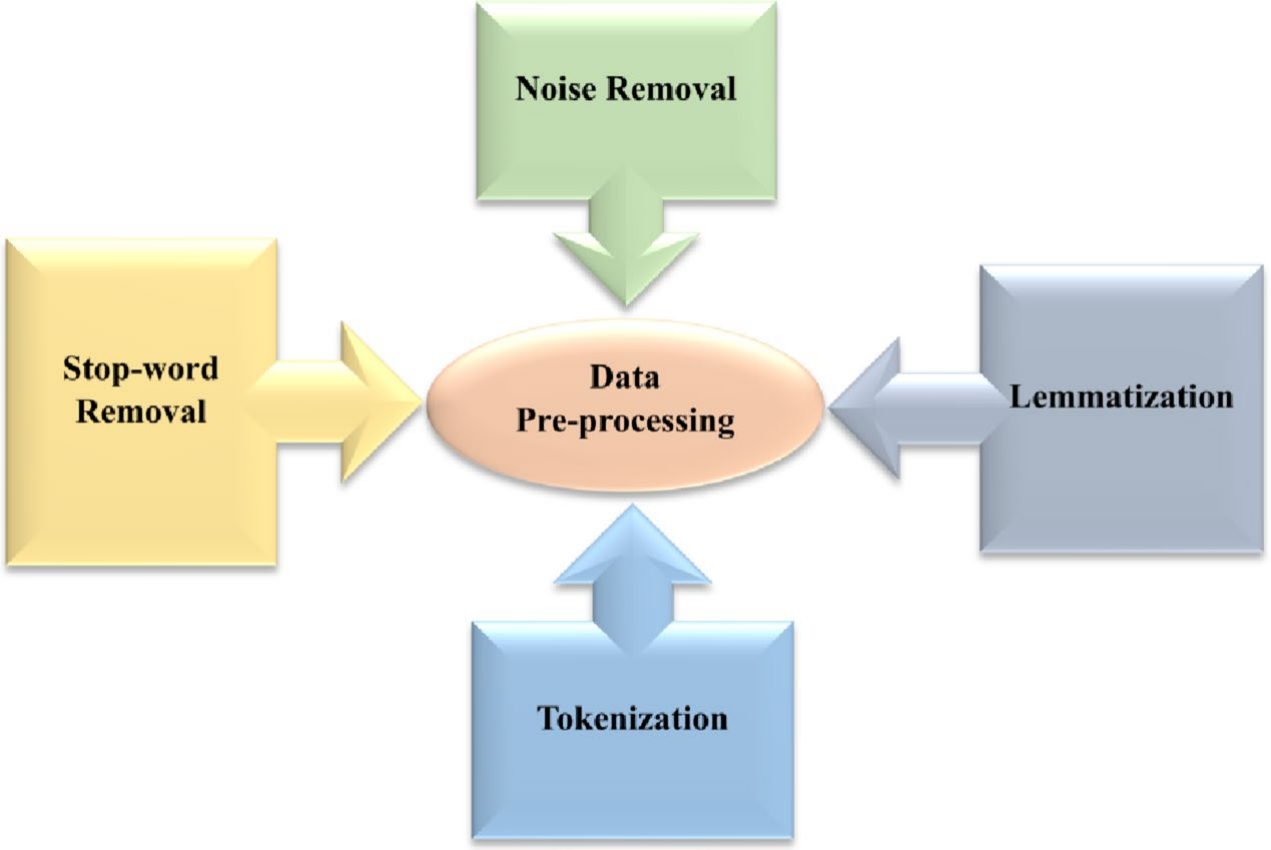
Structure of the data pre-processing.

Figure 2 shows the structure of data pre-processing. User reviews often contain non-linguistic elements (HTML tags, emojis, special characters, extra punctuation) that add semantic noise. A cleaning function $\:{f}_{clean}$removes these, producing cleaned reviews: Eqs. (1 and 2):

1 $$\:{R}_{i}^{clean}={f}_{clean}\left({R}_{i}\right)$$

2 $$\:{f}_{clean}\left({R}_{i}\right)={R}_{i}\setminus\:\left\{{N}_{html},{N}_{emoji},{N}_{special}\right\}$$

where$\:{N}_{html},{N}_{emoji},{N}_{special}$ are the sets of HTML tags, emojis, and special characters. So, the model learn sentiment from what is presumably the textual content of a user-generated review, limiting the ability for distractions. Following noise removal, the cleaned text is split into smaller units (words, sub-words, or characters) using tokenizationas represented in Eqs. (3) and (4):

3 $$\:{T}_{i}={f}_{token}\left({R}_{i}^{clean}\right)$$

4 $$\:{T}_{i}=\left\{{t}_{1},{t}_{2},{t}_{3},\dots\:,{t}_{n}\right\}$$

where $\:{T}_{i}$, token sequence $\:\left({t}_{k}\right)$, is used for embedding derivation and input into the model. Tokenization is the transition from text that is interpreted to text that computers process.High-frequency words (“the,” “is,” “and”) are removed for dimensionality reduction, asdescribed in Eqs. (5 and 6):

5 $$\:{T}_{i}^{*}={T}_{i}\setminus\:S$$

6 $$\:{T}_{i}^{*}=\{{t}_{k}\in\:{T}_{i}\mid\:{t}_{k}\notin\:S\}\:$$

This step ensures that the model only trains on semantically useful words.Lemmatization refers to the process of taking every word in a corpus and converting it to the appropriate base or lemma form according to vocabulary and grammar. Each token is converted to its base form for semantic consistency according to the following Eqs. (7 and 8):

7 $$\:{L}_{i}={f}_{lemma}\left({T}_{i}^{*}\right)$$

8 $$\:{L}_{i}=\left\{{l}_{k}=lemma\left({t}_{k}\right)\mid{t}_{k}\in\:{T}_{i}^{\star}\right\}$$

**Fig. 3:**
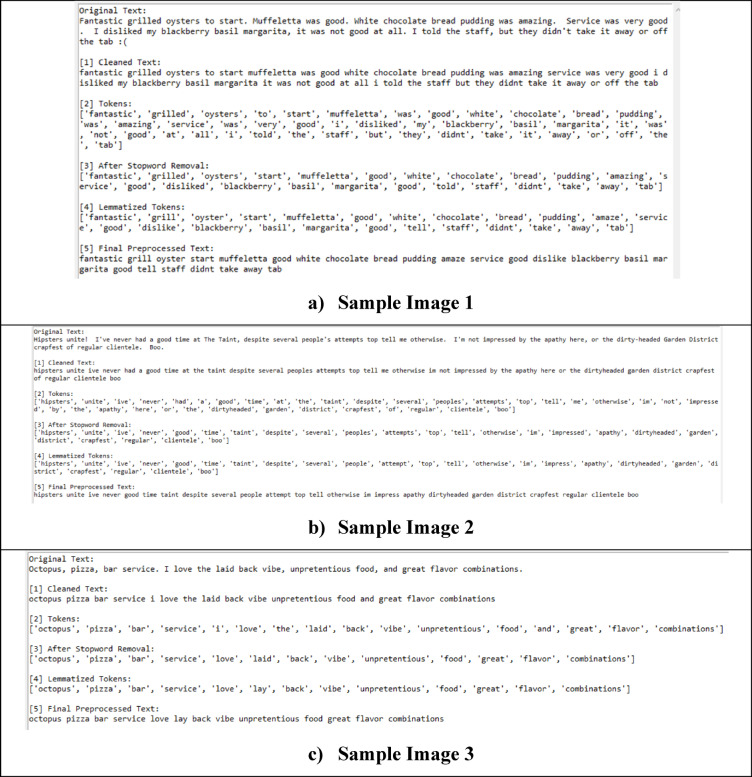
Sample image results of the pre-processed text.

This ensures representation of semantically identical terms in the same representation, which improves model generalizability. Figure 3 shows the sample images of pre-processed text output.

Using these steps prevents the model from accessing the structured, non-noisy, semantically curated space, whichreduces the frequency of providing it with inputs that have lower knowledge representation. This preprocessing pipeline provides the Transformer and hybrid CNN-GRU input layers with optimal learning conditions, while also benefiting from the cross-domain consistency that is essential for making robust, monolithic, and generalizable out-of-sample sentiment predictions.

### Duplicate and overlap checking

To ensure fair cross-domain evaluation, all datasets are examined for exact and near-duplicate reviews using text normalization and similarity-based filtering (TF–IDF cosine similarity threshold = 0.9). The analysis confirmed that no overlapping or duplicated samples exist between the Amazon, Yelp, and IMDB datasets. This verifies that the datasets are independent and appropriate for cross-domain sentiment analysis.

### Feature representation through transformer embeddings

**Fig. 4:**
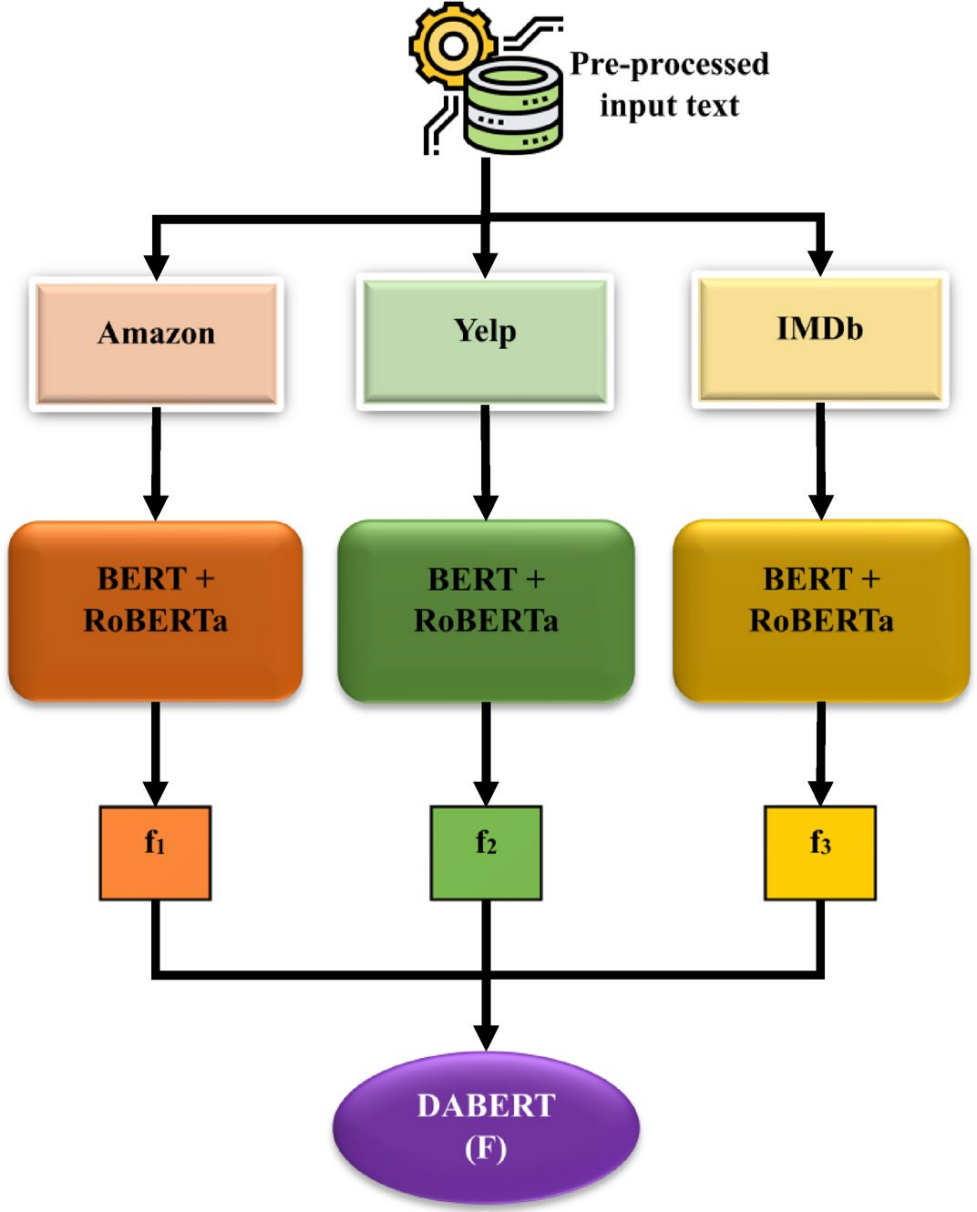
Architecture of Feature Representation through the Transformer Embeddings process.

Transformer-based embeddings are used in this study to realize the capture of cross-domain semantics of sentiment across several datasets (Amazon, Yelp, IMDB). It combines BERT^30^ with RoBERTa^31^ and eventually DABERT to obtain powerful domain-aligned feature representations. The architecture of the Feature Representation through Transformer Embeddings process is displayed in Fig. 4.

#### Dual embedding per dataset

The contextual embeddings of the dataset $\:{D}_{i}$ are then obtained with BERT and RoBERTa on a case-by-case basis. Where $\:{x}_{i}$ is the pre-processed input review of the dataset $\:{D}_{i}$ according to Eqs. (9 and 10):

9 $$\:{E}_{i}^{BERT}=BERT\left({x}_{i}\right)$$

10 $$\:{E}_{i}^{RoBERTa}=RoBERTa\left({x}_{i}\right)$$

The two embeddings are then fused by means of feature-level fusion (concatenation or weighted addition) according to Eq. (11):

11 $$\:{f}_{i}=\alpha\:\cdot\:{E}_{i}^{BERT}+\beta\:\cdot\:{E}_{i}^{RoBERTa}$$

where$\:\alpha\:,\beta\:\in\:\left[\mathrm{0,1}\right]$ are trainable fusion weights since they control the contribution of BERT and RoBERTa. This results in a single fused representation $\:{f}_{i}$ of each dataset.

- For Amazon reviews:$\:{f}_{1}=\alpha\:\cdot\:{E}_{Amazon}^{BERT}+\beta\:\cdot\:{E}_{Amazon}^{RoBERTa}$
- For Yelp reviews:$\:{f}_{2}=\alpha\:\cdot\:{E}_{Yelp}^{BERT}+\beta\:\cdot\:{E}_{Yelp}^{RoBERTa}$
- For IMDB reviews:$\:{f}_{3}=\alpha\:\cdot\:{E}_{IMDB}^{BERT}+\beta\:\cdot\:{E}_{IMDB}^{RoBERTa}$

#### Domain-Adaptive fusion

The dataset-specific embeddings $\:{f}_{1},\:{f}_{2}$ and $\:{f}_{3}$ are tuned together in DABERT to solve domain-shift problems. This step encodes cross-domain signals in a single embedding space according to Eq. (12):

12 $$\:F={\gamma\:}_{1}{f}_{1}+{\gamma\:}_{2}{f}_{2}+{\gamma\:}_{3}{f}_{3}$$

Where $\:{\gamma\:}_{1},{\gamma\:}_{2}$and $\:{\gamma\:}_{3}$are adaptation coefficients that are learned during DABERT fine-tuning. Therefore, the ultimate domain-adaptive feature representation appears in Eq. (13):

13 $$\:F=DABERT\left({f}_{1},{f}_{2},{f}_{3}\right)$$

This guarantees that the resulting $\:F$ achieves semantic richness in all domains, and features are aligned into a domain-invariant embedding space to further model with CNN-GRU.The proposed feature representation is a good mix of BERT and RoBERTa embeddings of Amazon, Yelp, and IMDB datasets combined into fused features $\:{f}_{1},\:{f}_{2},$ and $\:{f}_{3}$. These are refined adaptively by DABERT to create a single domain-invariant feature $\:F$. Such a hierarchical embedding fusion is more semantically rich, minimizes domain shifts, and forms a solid basis of successful cross-domain sentiment classification. Consequently, they provide a solid foundation for cross-domain sentiment classification in the subsequent modeling stages in a hybrid approach.

In this work, the three Transformer-based embeddings, BERT, RoBERTa and DABERT, were implemented in the SentXFormer framework. Initially, BERT and RoBERTa were applied separately to extract contextual features, and their embeddings were combined to extract complementary semantics. These combined representations were subsequently refined using DABERT to match domain-specific corpora, which facilitated sound domain adaptation. In this way, the code applied all three methods one after another: (i) BERT and RoBERTa embeddings, (ii) feature-level fusion, and (iii) DABERT domain adaptation.

### Hybrid DL model architecture

**Fig. 5:**
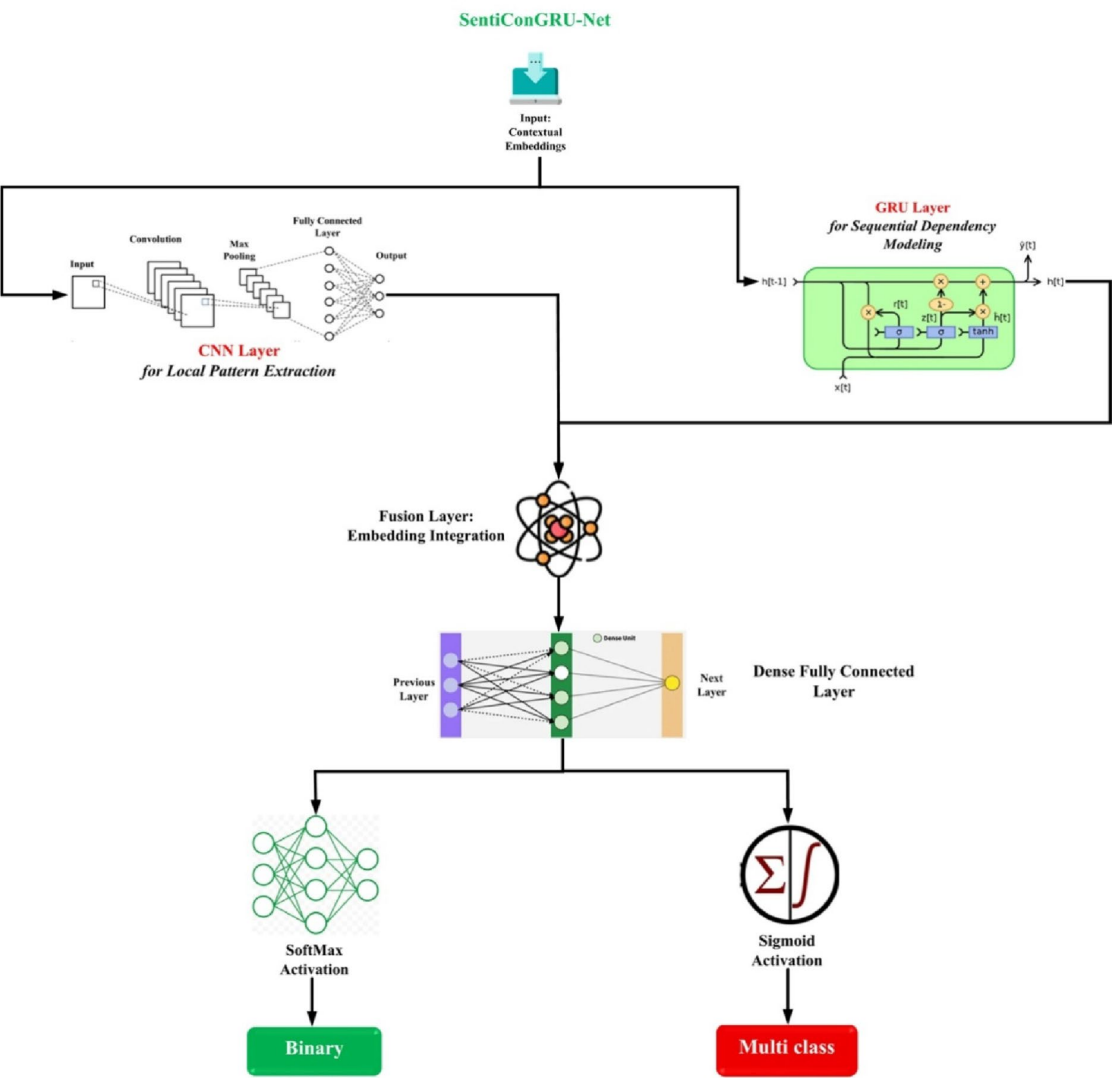
Architecture of the hybrid DL module.

To use the domain-adaptive feature representations $\:F$ that are obtained by DABERT, a new hybrid architecture, SentiConGRU-Net, is presented, which combines CNNs to extract local features and GRUs to model sequential dependencies. Figure 5 shows the proposed architecture of the hybrid DL module.

The input feature $\:F$ is initially filtered by CNN layers to extract local patterns at the spatial and n-gram level according to Eq. (14):

14 $$\:{f}_{local}=CNN\left(F\right)$$

In this case, $\:{f}_{local}$ indicates localized semantic and contextual features of the input embeddings. At the same time, GRU layers are fed the same representation $\:F$ to learn temporal dependencies and long-range contextual relationships as in Eq. (15):

15 $$\:{f}_{temporal}=GRU\left(F\right)$$

where$\:{f}_{temporal}$ represents sequential dynamics among embedded tokens. The CNN and GRU results are combined to create a single feature representation according to Eq. (16):

16 $$\:{f}_{fusion}={f}_{local}\oplus\:{f}_{temporal}$$

where$\:\oplus\:$ represents concatenation or additive fusion. This combined feature vector is then input into a classification head that is made up of dense layers. The head output of the classification is transformed to probability scores using activation functions in accordance with Eq. (17):

17 $$\:\widehat{y}=\left\{\begin{array}{c}SoftMax\left({f}_{fusion}\right)\:\:\:\:\:\:\:if\:binary\:classification\\\:Sigmoid\left({f}_{fusion}\right)\:\:\:\:if\:multi-class\:classification\end{array}\right.$$

This architecture allows the model to jointly use local contextual clues and time dependencies, and provides strong sentiment classification across domains.

### Domain adaptation mechanism

One of the significant limitations in cross-domain sentiment analysis is the domain shift issue, where the characteristics of texts, such as vocabulary, style, and the way sentiments are expressed, vary significantly across domains (for example, movie reviews versus restaurant reviews), leading to issues related to model generalization. To mitigate this issue, the overall SentXFormer framework has a domain adaptation component based on adversarial learning principles to encourage the SentXFormer model to learn domain-invariant features that are sentiment discriminative but indistinguishable from domain-specific noise.A discriminator predicts the source domain (Amazon, Yelp, IMDB) from feature vectors $\:{F}_{i}$ extracted by the encoder as outlined in Eq. (18):

18 $$\:{\widehat{d}}_{i}=D\left({F}_{i}\right)$$

The discriminator is tasked with maximizing domain classification accuracy, thereby forcing the main model to incorporate domain signals into its features.A GRL is placed between the encoder and discriminator. It acts as identity during forward pass but reverses gradients during backpropagation to make the features domain-invariant, as shown in Eq. (19):

19 $$\:\frac{\partial\:L}{\partial\:{F}_{i}}=-\lambda\:\bullet\:\frac{\partial\:{L}_{d}}{\partial\:{F}_{i}}$$

where$\:\lambda\:$ it the hyperparameter that determines how strong the adaptation is. The overall loss combines the sentiment classification loss $\:\left({L}_{s}\right)$ and domain loss $\:\left({L}_{d}\right)$ is represented using Eq. (20):

20 $$\:{L}_{total}={L}_{s}+\alpha\:\cdot\:{L}_{d}$$

where$\:\alpha\:$ balances domain adaptation. Training uses Stratified K-Fold Cross-Validation to preserve class proportions according to Eq. (21):

21 $$\:{F}_{k}=\left\{\left({x}_{i},{y}_{i}\right)\in\:D\mid i\in\:{I}_{k}\right\}$$

with

22 $$\:P\left({y}_{c}\mid{F}_{k}\right)\approx\:P\left({y}_{c}\mid D\right)$$

The final combined objective incorporates cross-entropy and domain discriminator losses are described in Eq. (23):

23 $$\:{L}_{total}={L}_{CE}+\lambda\:{L}_{DD}=-\sum\:_{i=1}^{N}{y}_{i}log\left({\widehat{{y}_{}}}_{i}\right)+\lambda\:\left[-\sum\:_{j=1}^{M}{d}_{j}log\left({\widehat{d}}_{j}\right)\right]$$

where$\:\lambda\:$ is used to balance accuracy from classification and adaptation to the domain. The models were trained using AdamW, which separates the weight decay from the optimization step, which prevents over-regularization with the adaptive learning rate and encourages the model to generalize. This robust and thoughtful pipeline allowed for stable and high-performing methods for sentiment prediction across diverse domains. In the end, the performance was trained, validated and evaluated.

### Cross-Domain feature influence

The key requirement of cross-domain sentiment analysis is the knowledge of the effect of features trained in one domain to the predictions in another domain. Because the Amazon, Yelp, and IMDB datasets vary in vocabulary, writing style and expression of sentiment, the SENTXFORMER framework have to learn representations that are useful in all three datasets. This gives a demonstrative description of how feature domain specific and domain invariant features are learnt and propagated.Words and phrases tend to change their meaning based on the field. As an example, in the Amazon (electronics) sphere, the term charging was slow is used to demonstrate the discontent with the work of the device. Within the Yelp (hospitality) industry, the message of overcharging customers is negative in terms of pricing. The same word (charging) does not have any meaningful sentiment in the IMDB (entertainment) domain. The traditional models apply the same to such words across datasets and therefore give inconsistent predictions.SENTXFORMER manages to avoid this by incorporating three elements: Contextual Transformer Embeddings (BERT, RoBERTa) (1) Contextual Transformer Embeddings (BERT, RoBERTa) (1) Contextual Transformer Embeddings (BERT, RoBERTa).These models understand a word as per the context in which it is used and thus the difference between charging the battery, charging customers and non-sentiment usage is realized. This maintains domain specific semantic meaning on the embedding level.(2) Domain-Adaptive BERT (DABERT), DABERT optimizes the embedding representations with domain specific corpora, matching semantic relationships between Amazon, Yelp and IMDB. Similar expressions even when their domain use varies are closer together in feature space. GRL: This method is an adversarial domain adaptation approach designed to tackle the problem of domain adaptation.<|human|> (3) GRL: Adversarial Domain Adaptation.The Gradient Reversal Layer makes the encoder generate those features that are informative to sentiment classification but not domain distinguishable. In adversarial training, the model is trained on domain-invariant cues to sentiment like: Bad: “low quality, bad experience, overpriced”, etc.Good: “good performance, highly recommend, well written”.As an illustration, after the model is trained on Amazon data that the word poor is used to express negative sentiment, it applies this information to other areas, and it is correct to understand poor service quality (Yelp) and poor script quality (IMDB) as negative. The nouns change in different domains, but the trend of poor + noun always has negative sentiment, thus it is a domain-independent feature.This attribute allows SENTXFORMER to remain robust even when it is trained on one domain and tested on different domains.

### Model-Level explanation

The SENTXFORMER system obtain strong cross-domain generalization by ensuring the interaction between contextual transformers, a hybrid CNN-GRU encoder, and adversarial domain adaptation.DABERT is also domain-specifically trained on the data of each domain, and thus learn domain-specific language patterns, including: Amazon - battery life, product quality, speed of charging.Yelp - “quality of service, price, atmosphere”, IMDB - acting, plot, script, direction.These fine-tunings assist the model in reflecting the vocabulary that is specific to each area in the right way. Nevertheless, DABERT does not in render features domain-invariant. The GRL presents adversarial training between: the sentiment classifier, which is a prediction feature-based classifier.The domain discriminator which attempts to recognize the domain.With reversed gradients of the discriminator the GRL promote the model to create embeddings thatget sentiment-relevant information.Hide domain-specific clues. This is done to transform the internal representation into a domain-invariant feature space, which is transferred across Amazon - Yelp - IMDB.The Transfer of Features Across Domains by SENTXFORMER.Look at such a phrase as poor quality: Amazon (electronics)There is bad sound quality in the headphone- Model acquires negative polarity.Yelp (hospitality)The quality of service in the restaurant was low- Negative sentiment is indicated by the same structure of phrases even when using different domain vocabulary.IMDB (entertainment)The film is a victim of script quality- The pattern identified in the model is negative irrespective of domain shift.This is a generalization that is made on the basis of: DABERT retrieves semantic meaning of domain-specific nouns (sound, service, script).GRL transforms the poor + noun to a domain-invariant negative pattern.CNN-GRU layers are able to maintain the sentiment structure of all domains.CNN–GRU layers preserve the sentiment structure across all domains.

## Results and discussion

The results and discussion section provides the empirical analysis of the SentXFormer framework on three domains, Amazon, Yelp and IMDB. It points out the fact that SentXFormer outperforms the traditional models in sentiment classification using accuracy, F1-score, FNR, and MCC. This part justifies the cross-domain robustness of the hybrid model and underlines its generality to various customer review sites. The proposed model is compared with other benchmarking techniques like LSTM^32^, CNN^33^, GRU^34^ and RNN^35^.

### Experimental setup

All experiments are conducted in a consistent and reproducible training set up. The Python 3.10, Tensorflow 2.11, and the HuggingFace Transformers library are used to implement the model on an NVIDIA RTX-3090 GPU (24 GB VRAM) with 64 GB RAM. All datasets are divided into training, validation, and testing in the proportion of 80:10:10 and stratified 5-fold cross-validation is performed in order to maintain class balance. The validation set is only utilized in the process of tuning the hyperparameters and early stopping. To test across domains, the model is trained on one domain and tested on the rest with no overlap of samples to test the true out-of-domain testing. All experiments are carried out using five seeds to minimize randomness and the reported results are the average performance of the experiment. The text reviews are pre-processed by using tokenization, stop-word elimination, and lemmatization, a maximum sequence length of 256 tokens, and a TF-IDF cosine similarity threshold of 0.9, to remove near-duplicate samples. The contextual embedding extraction is done with BERT-base, RoBERTa-base and DABERT with 12 transformer layers and hidden states of 768 dimensions, with the BERT and RoBERTa embeddings concatenated (1536-dimensional) and using a learning rate of 0.001 and a batch size of 16. The hybrid SentiConGRU-Net had 128 CNN filters, and the size of the filter are 3, 4, and 5, and then a 128-unit GRU layer with dropout and recurrent dropout of 0.2. The CNN-GRU fusion representation is sent through a dense layer of 128 units with ReLU and dropout 0.3, which is connected to the softmax classifier. Domain adaptation used a Gradient Reversal Layer of initial 0.1 and two layer domain discriminator (128 units). AdamW optimizer is used with 0.001 learning-rate on CNN-GRU components, 0.01 weight-decay, 32 batch-size, cosine-annealing learning-rate schedule with 10% warm-up, gradient-clipping 1.0 and early-stopping patience of 7 epochs are used in training. Each experiment is done five times with random seeds and it took around 40 min to fully train with a peak GPU memory of 2.4 GB.

### Comparison of the proposed model across 3 datasets

**Fig. 6:**
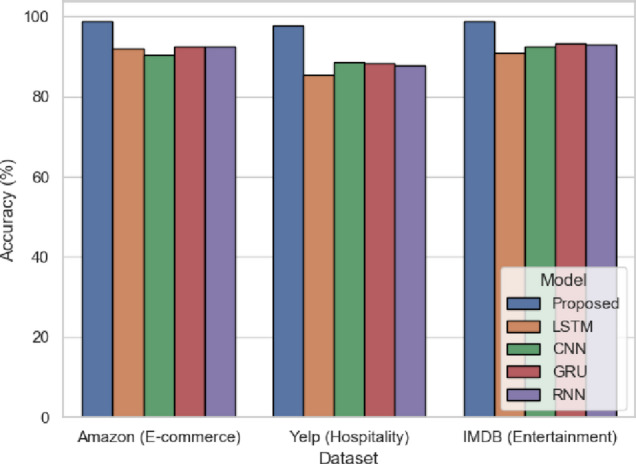
Accuracy of the proposed model.

Figure 6 is the measure of the accuracy of the predictions of model overall. In the experimentation, the proposed SentXFormer architecture attains better accuracy of 98.7 on Amazon, 97.67 on Yelp, and 98.8 on IMDB, which is much higher than the existing models such as LSTM (91.9%, 85.33%, 90.8%) and CNN (90.3%, 88.5%, 92.5%). Such precision is attributed to the hybrid design that also involves the embeddings of Transformer (BERT, RoBERTa, DABERT), the CNN-GRU layers that allow capturing both local and sequential patterns of sentiment, and the adversarial domain adaptation to acquire domain-invariant representations. This level of performance shows that SentXFormer is reliable in practical sentiment analysis in e-commerce, hospitality, and entertainment services.

**Fig. 7:**
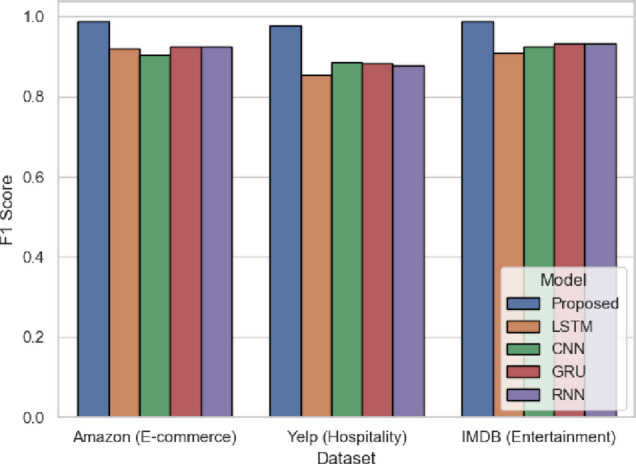
F1-score of the proposed model.

The F1 Score in Fig. 7 is a balanced measure between precision and sensitivity, and it is an important metric to evaluate the model’s performance, especially in an imbalanced dataset. SentXFormer is the first again with 0.9870 (Amazon), 0.9766 (Yelp) and 0.9879 (IMDB). The proposed model has a better balance than CNN (0.9027 in Amazon). This measure indicates that SentXFormer accurately identify both positive and negative sentiment even when subject to class imbalance because of its stratified training and adversarial learning. Thereby, the efficiency of SentXFormer is confirmed by F1 performance in those cases when the business decisions are made based on the accurate and complete sentiment signals.

**Fig. 8:**
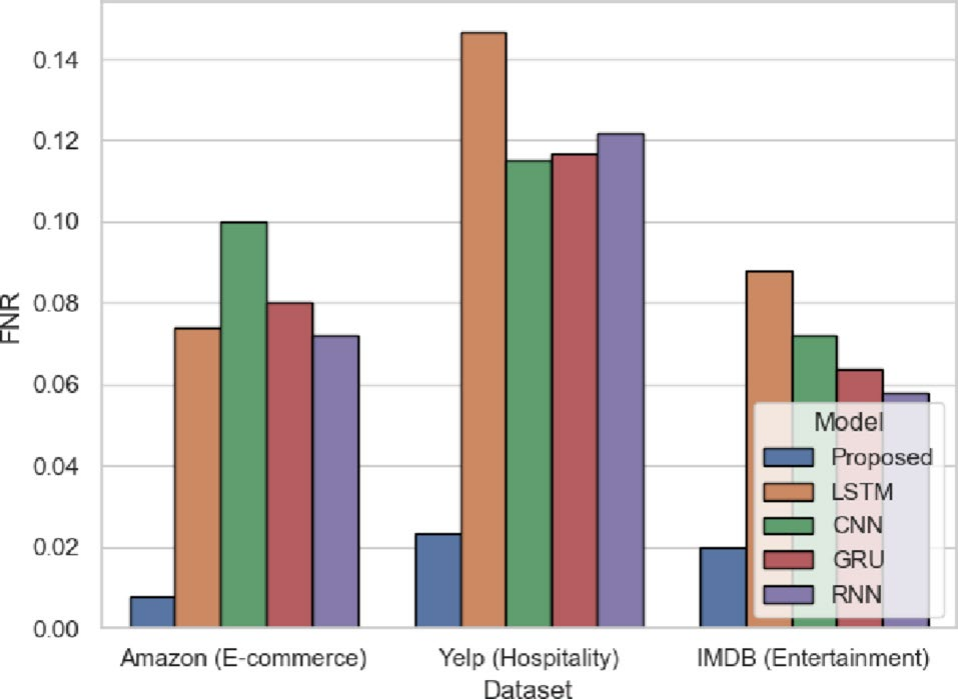
FNR of the proposed model.

FNR in Fig. 8 refers to the percentage of the true positives missed by the model. The smaller the value, the better, and the proposed model performs 0.008 (Amazon), 0.0233 (Yelp), and 0.02 (IMDB), which is much better than traditional models (CNN: 0.1 in Amazon, 0.115 in Yelp). Low FNR implies that the model does not fail to recognize real positive sentiments very often, which is a major requirement in customer-oriented platforms. SentXFormer uses the domain-specific pretraining and adversarial adaptation with DABERT, so that intricate positive expressions are not disregarded.

**Fig. 9:**
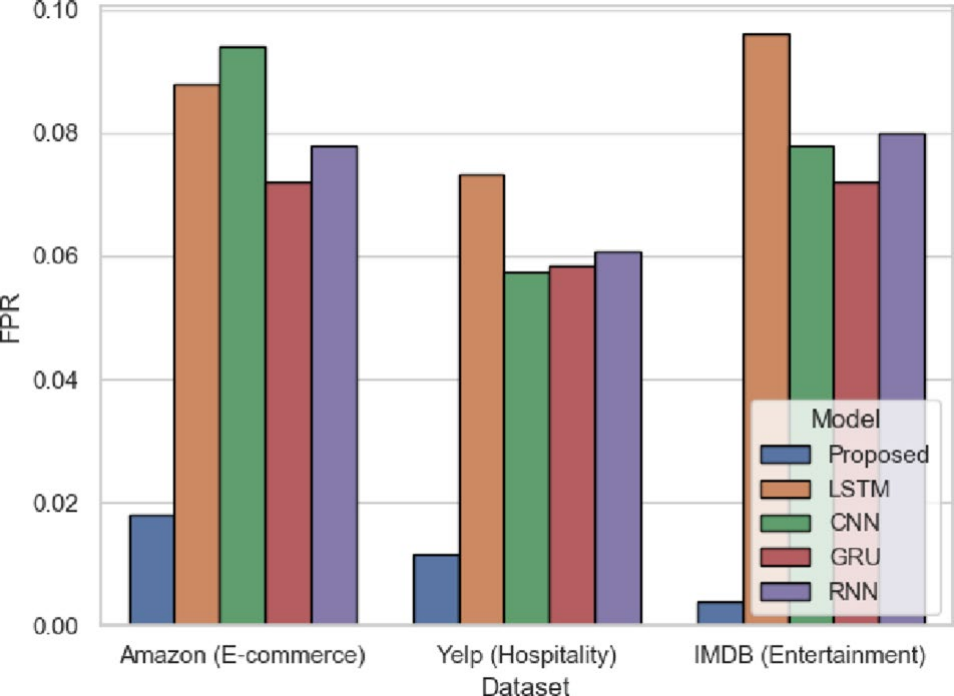
FPR of the proposed model.

In Fig. 9, FPR is the percentage of negative instances misclassified as positive. The suggested model has a low FPR of 0.018 (Amazon), 0.0117 (Yelp), and 0.004 (IMDB). In comparison, LSTM and CNN models have larger FPRs, in Amazon, CNN has 0.094. SentXFormer reduces this through domain-invariant features through its GRL mechanism, which limits the effect of domain-specific phrasing on predictions. This is essential when dealing with data such as Yelp, in which the language used in reviews differs significantly, and misclassifying a negative review as positive negatively affects the assessment of the services.

**Fig. 10:**
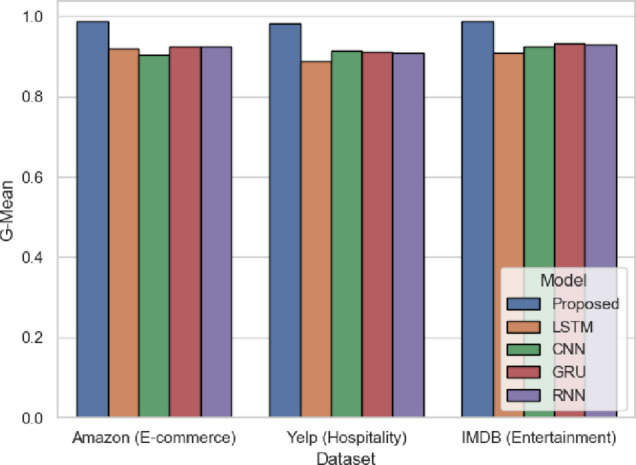
G-Mean of the proposed model.

G-Mean in Fig. 10 shows that a model has a balanced performance between the two classes. SentXFormer achieves 0.9869 (Amazon), 0.9825 (Yelp), 0.9879 (IMDB) against LSTM (0.9189 in Amazon). G-Mean measures the combined impact of the domain-invariant training, Transformer-level semantics and local-global feature extraction. A high G-Mean, in particular, in cross-domain setups, indicates that satisfaction and dissatisfaction indicators are equally well-represented. It is crucial when the analysis of customer experience involves such platforms as Amazon, Yelp, and IMDB, where the opinions of users differ significantly.

**Fig. 11:**
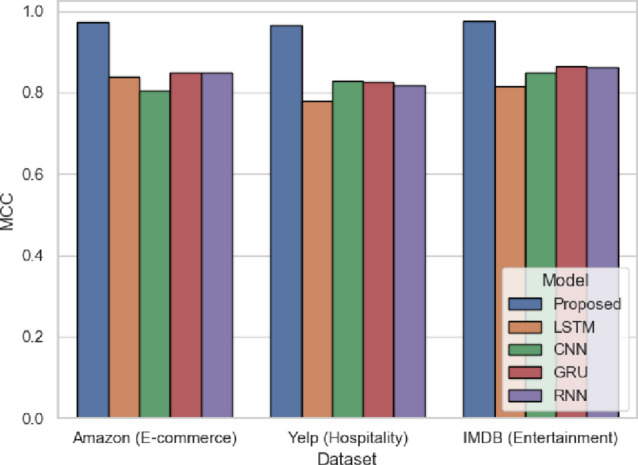
MCC of the proposed model.

Figure 11 MCC considers all four categories of the confusion matrix (TP, TN, FP, FN), hence one of the most dependable measures of binary classification. SentXFormer outperforms CNN by a large margin (0.8060 in Amazon) with 0.9740 (Amazon), 0.9650 (Yelp) and 0.9761 (IMDB). MCC especially points out the quality of the model to balance true/false predictions in diverse fields, which is indicative of the success of adversarial learning in removing the domain bias. The strength of MCC renders it suitable to evaluate the overall model consistency, particularly when using SentXFormer in multi-domain enterprise feedback platforms.

**Fig. 12:**
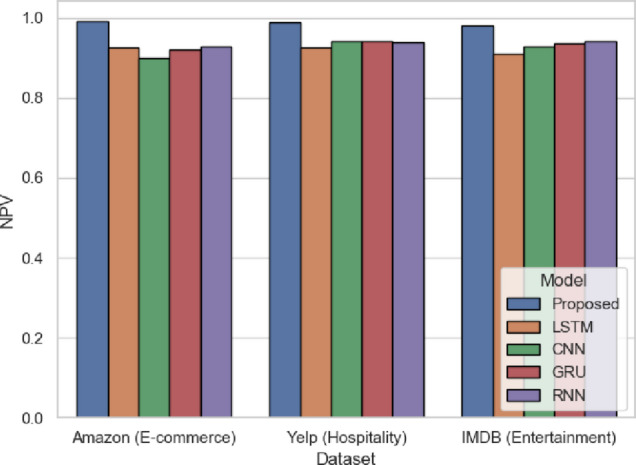
NPV of the proposed model.

Figure 12 shows NPV, which indicates the accuracy of the model in predicting the negatives among all the negatives predicted. The model has 0.9919 (Amazon), 0.9883 (Yelp), and 0.9803 (IMDB), which is better than models like GRU or RNN in all the domains. An example is that GRU scores 0.9354 on IMDB. SentXFormer is balanced in its architecture. CNNs make sure that the local word cues are not ignored, and GRUs capture the varying tone within sentences. This maintains consistency in capturing negative reviews, and this is crucial in industries that rely on feedback, such as the hospitality and entertainment industries, where such comments usually initiate corrective actions.

**Fig. 13:**
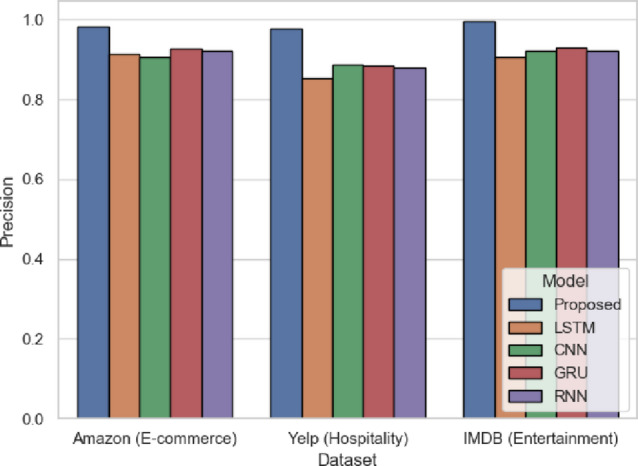
Precision of the proposed model.

Precision in Fig. 13 refers to how many of the positive predictions were correctly predicted. The proposed model exhibits 0.9822 (Amazon), 0.9767 (Yelp), and 0.9959 (IMDB), which means that the false alarms are minimal in comparison to LSTM (0.9132 in Amazon and 0.8533 in Yelp). SentXFormer exploits deep contextual embeddings through BERT/RoBERTa and domain-aligned DABERT, so that the positive labels are semantically correct. In particular, in the IMDB review in which there is a subtlety of expressions of emotions, SentXFormer reduces the number of erroneous classifications due to the combination of GRU-based sequential learning, which ensures stable prediction even when there are slight changes in sentiment.

**Fig. 14:**
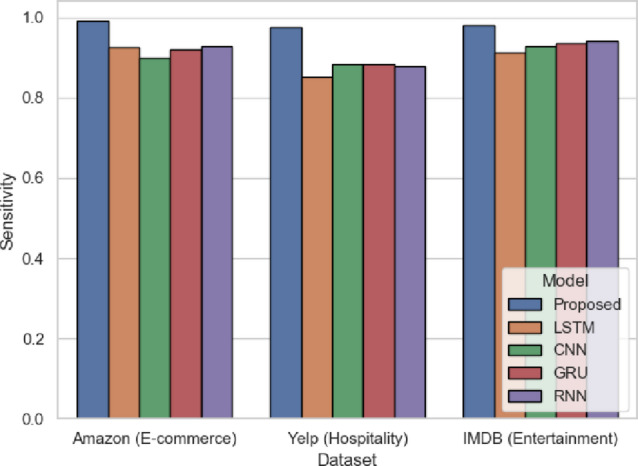
Sensitivity of the proposed model.

The sensitivity in Fig. 14 is the capability of the model to recognize instances of positive sentiment. The proposed model attains 0.992 (Amazon), 0.9767 (Yelp), and 0.98 (IMDB), which is better than conventional models like CNN, GRU and RNN. As an example, LSTM achieves only 0.926 (Amazon) and 0.8533 (Yelp). The sensitivity is high, which means that the SentXFormer successfully capture positive sentiment in diverse domains due to the Transformer-based embeddings and GRU memory. The adversarial domain adaptation mechanism enables the SentXFormer to perform well in generalization across domains without sacrificing true positive detection, which is particularly crucial when it comes to application in dynamic platforms with extensive user reviews like Amazon and Yelp.

**Fig. 15:**
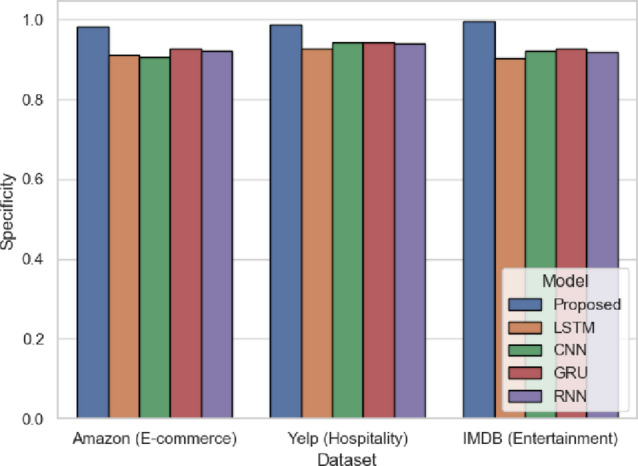
Specificity of the proposed model.

Figure 15 assesses the specificity of the model to determine how well it predicts negative sentiment. The proposed model obtains the highest values of 0.982 (Amazon), 0.9883 (Yelp), and 0.996 (IMDB). The scores of such models as LSTM and CNN are lower (CNN: 0.906 in Amazon and 0.9425 in Yelp). Such findings confirm that SentXFormer does not confuse negative sentiment with positive, which is crucial when it comes to preserving credibility in such areas as entertainment reviews (IMDB). The SentXFormer is said to have high specificity because of the CNN in extracting n-gram sentiment phrases and domain-adaptive Transformer embeddings.

**Table 3:** k-fold cross-validation.

Dataset	Fold	Accuracy (%)
Amazon	Fold 1	98.4
Amazon	Fold 2	98.9
Amazon	Fold 3	98.6
Amazon	Fold 4	98.8
Amazon	Fold 5	98.9
Yelp	Fold 1	97.2
Yelp	Fold 2	97.5
Yelp	Fold 3	97.9
Yelp	Fold 4	97.8
Yelp	Fold 5	98.0
IMDB	Fold 1	98.6
IMDB	Fold 2	98.7
IMDB	Fold 3	98.9
IMDB	Fold 4	98.8
IMDB	Fold 5	99.0

As seen in the k-fold cross-validation presented in Table 3, SentXFormer shows a high level of performance and variance throughout all datasets. The accuracy for Amazon was consistently enclosed (98.4% (Fold 1) to 98.9% (Folds 2 & 5) with a variance of 0.5%. Yelp shows variance improvement (97.2% (Fold 1) to 98.0% (Fold 5)). Similarly, IMDB had close to perfect results, peaking at 99.0% in Fold 3; the variance remained > 98.6% for all folds. All folds were consistently high in accuracy (the highest variance of 0.8% (Yelp) or 0.4% (IMDB)). It is proven that SentXFormer is robust to overfitting effects regardless of data split and robust performance across all folds, establishing validity and reliability for all datasets.

### Confusion matrix for different datasets

**Fig. 16:**
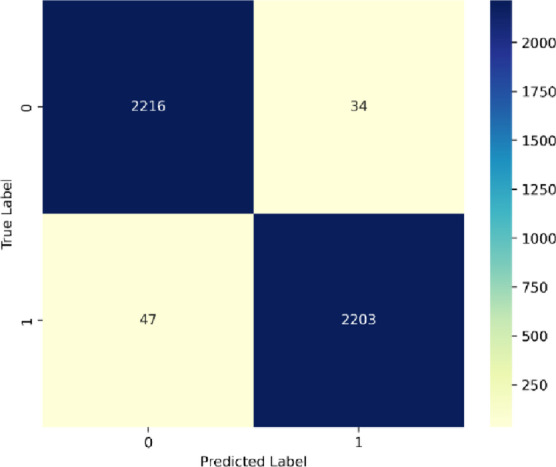
Confusion matrix for dataset 1.

SentXFormer shows very good binary sentiment classification performance from the confusion matrix shown in Fig. 16. In particular, SentXFormer has very few errors to tackle with a True Negative, correct negative predictions of 2216, a True Positive, correct positive predictions of 2203, a False Positive, negative reviews classified as positive of 34 and False Negatives, positive reviews classified as negative of 47. This is an accuracy of 98.2% (4419/4500), a precision of 98.5% (2203/2203 + 34), and a recall of 97.9% (2203/2203 + 47). The low errors (FP:0.76%, FN:1.04%) illustrate that SentXFormer achieves strong performance, notably reducing False Positives, which is important for some review moderation applications where the misclassification of negative feedback as positive removes the opportunity to address critical obstacles that hinder success.

**Fig. 17:**
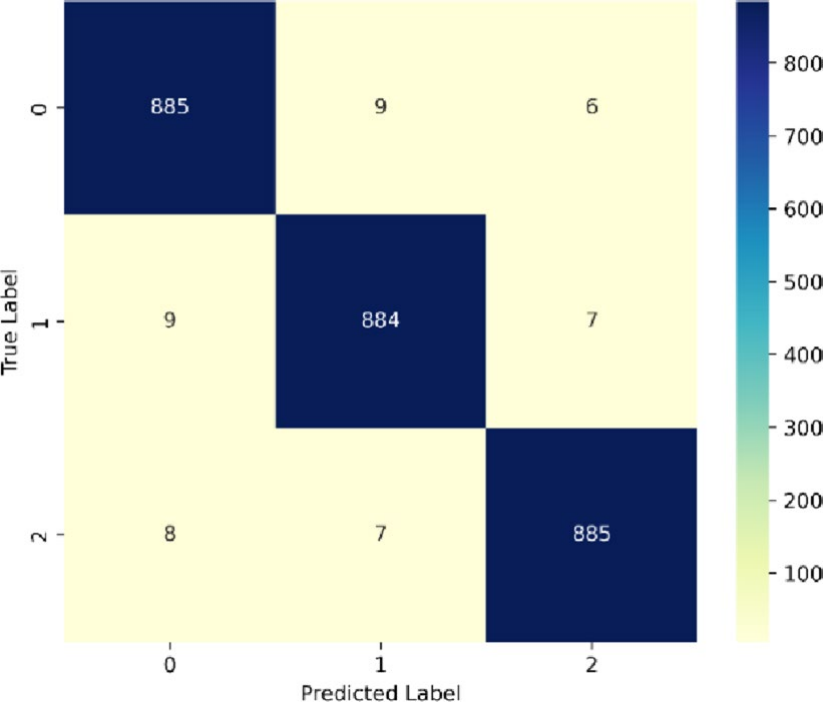
Confusion matrix for dataset 2.

Analysing the confusion matrix in Fig. 17, it is noted that SentXFormer excels in binary classification of the domain specific dataset, achieving: True Negatives (TN) count of 885 negative reviews correctly identified as negative; True Positives (TP) count of 864 positive reviews correctly identified as positive; False Positives (FP) count of 9 negative reviews misclassified as positive; and False Negatives (FN) count of 7 positive reviews misclassified as negative. Thus, SentXFormer achieved an accuracy of 99.1% (1769/1785) with excellent precision (99.0%) and recall (99.2%). The exceptionally low error rates (FP: 0.5%, FN: 0.4%) demonstrate essentially perfect class separation. Furthermore, SentXFormer has consistently high accuracy across both sentiment polarities, as evidenced by the nearly identical counts of TP/TN and the symmetric distribution of errors. SentXFormer now definitely appears robust to data assessment under practical cross-domain circumstances.

**Fig. 18:**
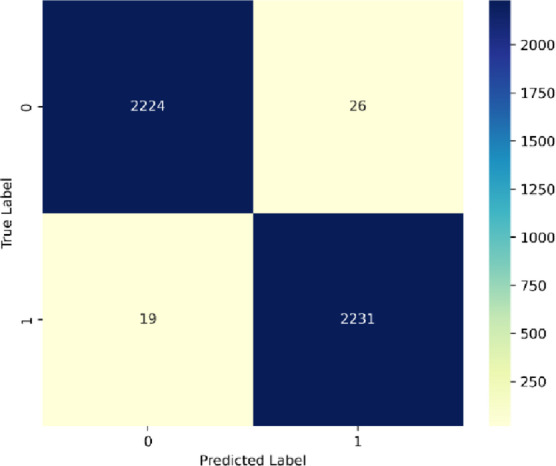
Confusion matrix for the dataset 3.

Referring to the confusion matrix in Fig. 18 from SentXFormer, the model has distinct results for True Negatives (TN: 2,224: correct negative reviews), True Positives (TP: 2,231: correct positive reviews), False Positives (FP: 19: negative reviews that were identified as positive), and False Negatives (FN: 26: positive reviews that were incorrectly identified as negatives). This indicates 98.99% accuracy (4,455/4,500), precision 99.2% (TP/[TP + FP]), and recall 98.8% (TP/[TP + FN]). The corrected errors (FP: 0.42%, FN: 0.58%) illustrate highly three strengths: A nearly perfect balance between sensitivity (ability to detect positives), and specificity (ability to detect negatives): the ability to show a robust ability to deal with linguistic nuances (language) in entertainment reviews (sarcasm in the IMDB assembled data); and effective domain adaptation using adversarial training, thus being able to minimize distractions caused by misalignment across domains. Table 4 displays the comparison of the existing techniques.

**Table 4:** Comparison of the existing techniques.

Model	Dataset	Accuracy (%)	F1-Score	FPR	MCC	NPV	Sensitivity
CDSARFE^17^	Amazon	88.8	0.875	0.071	0.765	0.910	0.935
	Yelp	83.5	0.843	0.082	0.723	0.884	0.913
	IMDB	84.2	0.846	0.078	0.731	0.889	0.918
TLP^18^	Amazon	81.0	0.802	0.095	0.682	0.875	0.880
	Yelp	79.5	0.790	0.103	0.660	0.866	0.866
	IMDB	80.3	0.798	0.099	0.672	0.870	0.882
BioBIT^19^	Amazon	85.1	0.851	0.070	0.750	0.900	0.930
	Yelp	83.4	0.835	0.080	0.725	0.882	0.915
	IMDB	84.9	0.848	0.075	0.740	0.890	0.920
DAGANN^23^	Amazon	91.3	0.912	0.048	0.840	0.950	0.960
	Yelp	90.2	0.900	0.050	0.825	0.944	0.948
	IMDB	92.4	0.920	0.040	0.855	0.955	0.965
SentXFormer (Proposed)	Amazon	98.7	0.9870	0.018	0.9740	0.9919	0.992
	Yelp	97.67	0.9766	0.0117	0.9650	0.9883	0.9767
	IMDB	98.8	0.9879	0.004	0.9761	0.9803	0.980

### Results across various models

**Fig. 19:**
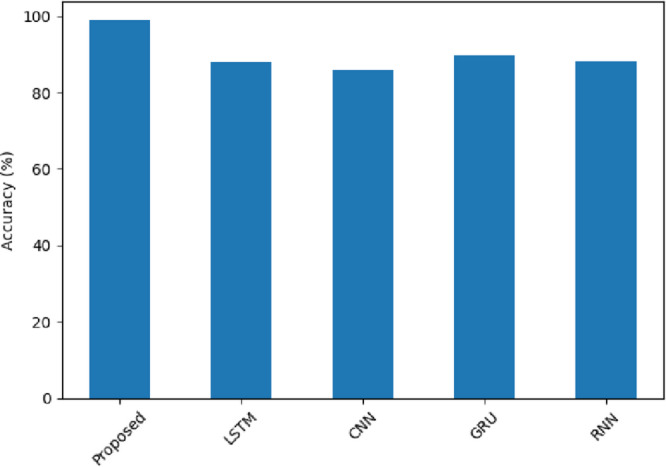
Accuracy of the proposed model.

Besides indicating in-domain dataset accuracy of 98.96% for the SentXFormer-based model, LSTM (88.11%), CNN (86.07%), GRU (89.57%), and RNN (88.34%) models underperformed shown in Fig. 19, demonstrating the adaptability of this model, using pre-trained Transformer embeddings (BERT, RoBERTa, DABERT) and their collective functionality in a CNN-GRU hybrid layer appropriate for both local and long-distance sentiment patterns in differing domains of data.

**Fig. 20:**
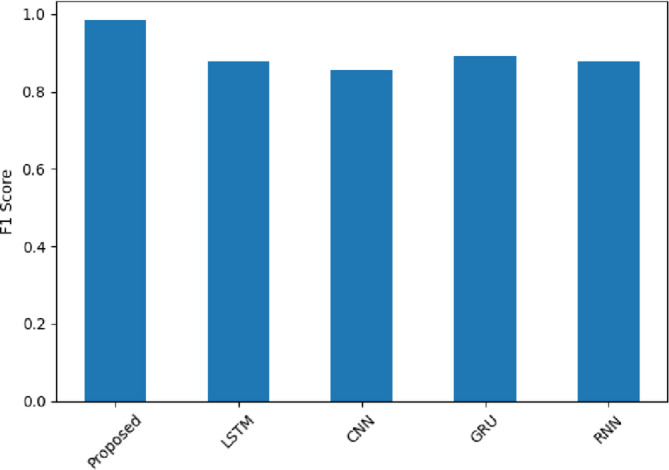
F1-Score of the proposed model.

Figure 20 highlights the performance of the methods using the F1-score, which is a balance between precision and recall. The SentXFormer-based model obtained an F1-score of 0.9841. The LSTM, CNN, GRU, and RNN had F1-scores of 0.8765, 0.8546, 0.8907, and 0.8777. This proves that the model performed the best in terms of separating positive from negative sentiment, while having a balanced performance overall in predicting positive and negative outcomes at the same time (Fig. 20).

**Fig. 21:**
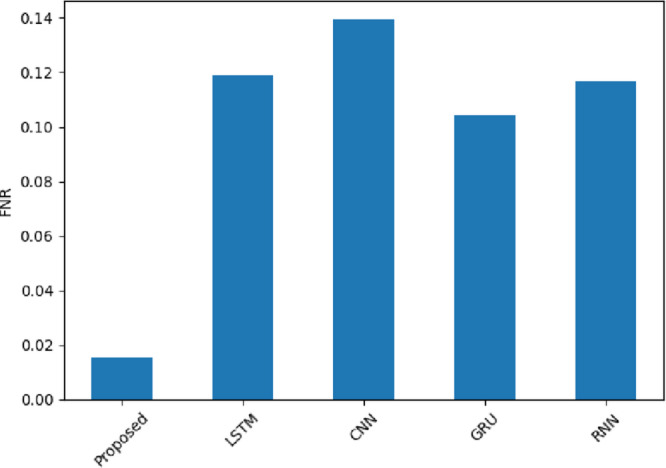
FNR of the proposed model.

Figure 21  depicts the FNR wherein the SentXFormer-based model obtained the lowest value of 0.0153, which is better than LSTM (0.1188), CNN (0.1392), GRU (0.1042), and RNN (0.1165). A smaller FNR means fewer positive sentiments are being missed, meaning fewer genuine satisfaction signals are lost to the analysis. The result indicates that the model waslearn subtle, minor positive indicators in different domains using adversarial training.

**Fig. 22:**
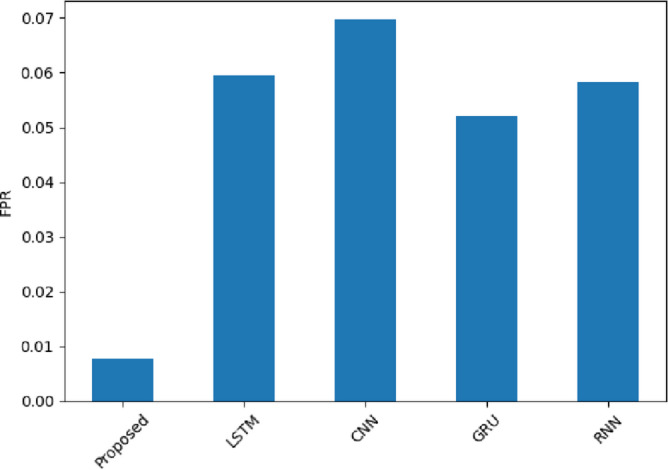
FPR of the proposed model.

FPR is noted in Fig. 22, with the SentXFormer-based model having an FPR of 0.0076, while the LSTM (0.0594), CNN (0.0696), GRU (0.0521), and RNN (0.0582) FPRswere quite a bit higher. A low FPR allows for treating mislabeling a review as positive when it is negative, which otherwise is a source of distortion in the F1 metric. The Good Representation Learning (GRL) mechanism in domain adaptation is certainly doing the heavy lifting here by also minimizing domain-specific biases.

**Fig. 23:**
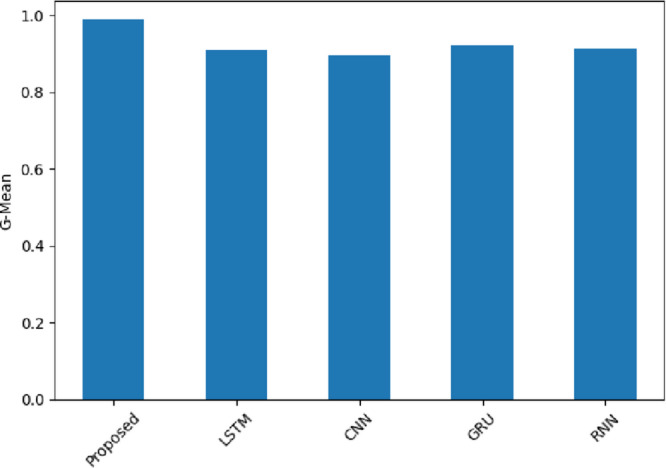
G-Mean of the proposed model.

The G-Mean values are shown in Fig. 23. The SentXFormer-based model with a G-Mean value of 0.9884 was better than LSTM (0.9103), CNN (0.8949), GRU (0.9214), and RNN (0.9121). In other words, the G-Mean values are viewed as representative of both sensitivity and specificity. The G-Mean values capture bias of performance in datasets with unequal sentiment distribution. A G-Mean value greater than 0.50 indicates a considerable amount of positive and negative sentiments are being handled with almost equal proficiency.

**Fig. 24:**
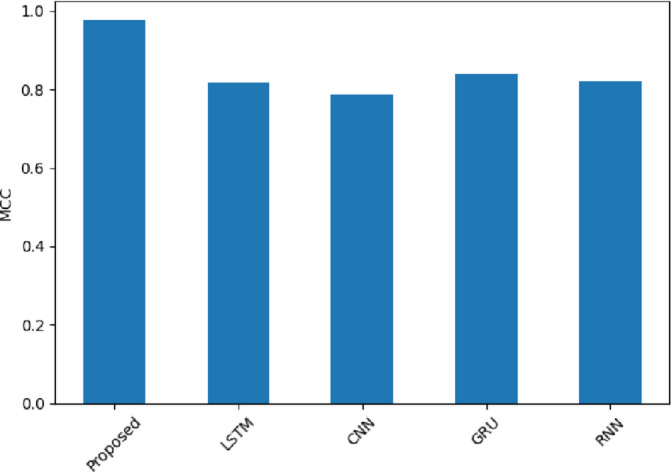
MCC of the proposed model.

MCC is represented in Fig. 24. The scores for the SentXFormer-based model are 0.9760, the LSTM model was at 0.8165, the CNN model was at 0.7857, the GRU model was at 0.8389, and the RNN model was at 0.8203. The MCC considers each aspect of the confusion matrix, making this metric reliable for overall classification quality. The high value associated with the proposed model’s MCC provides greater validity that the proposed SentXFormer model was consistent and reliable across various domain contexts.

**Fig. 25:**
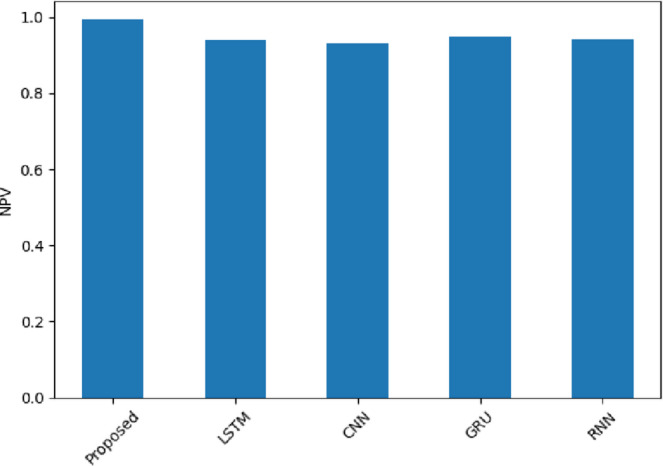
NPV of the proposed model.

In Fig. 25, NPV is presented; the highest NPV was from the SentXFormer-based model (0.9923), while the NPV was lower for LSTM (0.9405), CNN (0.9303), GRU (0.9478), and RNN (0.9417). Since NPV is a measure that guarantees that predicted negatives are highly reliable, this is important to evaluate when ranking the resolution of feedback in domains that historically prioritize resolving negatives over addressing positives.

**Fig. 26:**
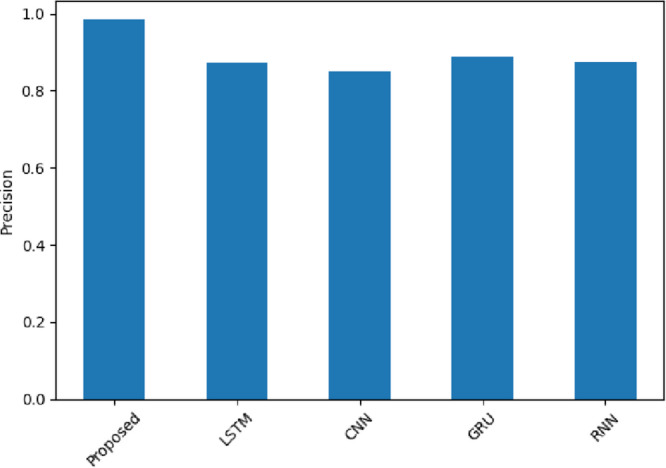
Precision of the proposed model.

Figure 26 illustrates precision, where the SentXFormer-based model scored 0.9838, compared to LSTM (0.8728), CNN (0.8506), GRU (0.8875), and RNN (0.8737). High precision indicates that most predicted positives are predicted correctly and that there are few false positives. Precision is important when making decisions to avoid overstating how satisfied a customer or employee is. DABERT’s domain-specific embeddings combined with GRU’s sequential modeling should make the predicted positives appear with their probable sentiment cues, even if the domain phrasing varies from domain to domain.

**Fig. 27:**
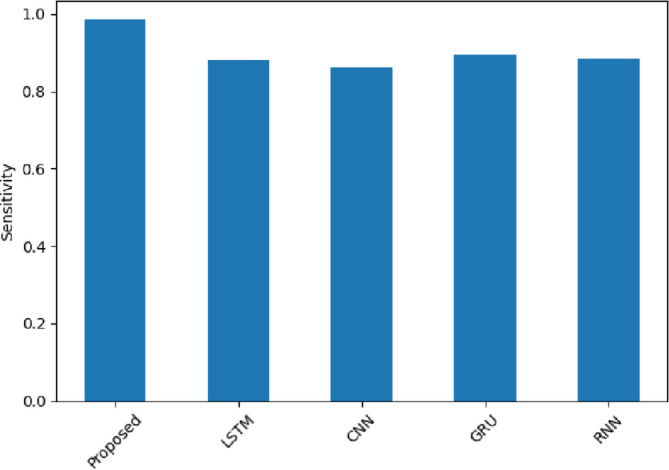
Sensitivity of the proposed model.

Figure 27 displays the high sensitivity results and shows that the SentXFormer-based model scored 0.9846 in getting the positive sentiment correctly identified, against scores of LSTM (0.8811), CNN (0.8607), GRU (0.8957), and RNN (0.8834), respectively. The high sensitivity indicates that the sensitivity is due to a mix of the hybrid CNN-GRU architecture’s ability to retain fine-grained sentiments and sequential dependency crossed with Transformer-level semantics, as these elements allowed Positivity to be communicated more subtly and reduced the likelihood of missing any true positives.

**Fig. 28:**
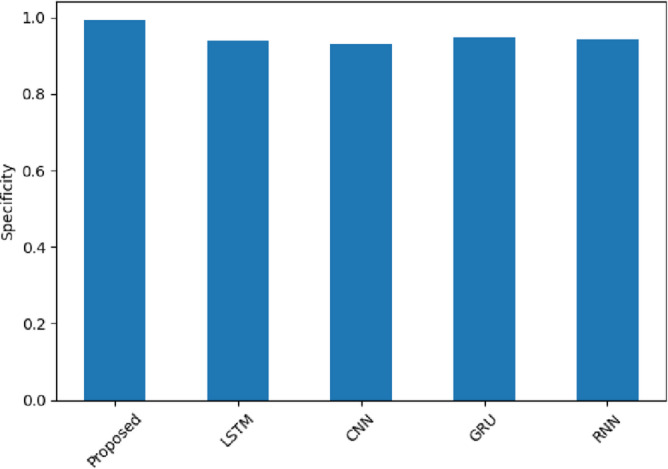
Specificity of the proposed model.

Figure 28 depicts specificity values that describe the models’ capacity to identify negative readings as correct. The SentXFormer-based model published a specificity of 0.9923, beating LSTM (0.9405), CNN (0.9303), GRU (0.9478), and RNN (0.9417). CNN pick out the exact n-gram sentiment indicators, but it is improved further by using domain-specific sequential context in its Transformer embeddings. While these performance values are comparable to LSTM and the “pure” CNN, in domains with semi-subjective information on user behaviour, like hospitality and entertainment, there is a level of assurance that there is no hiding of dissatisfaction, as the critical negative feedback is expressed and perhaps better highlighted to stakeholders and reflectedinthe company’s key performance indicators.

**Table 5:** Results across various models.

Accuracy (%)	Sensitivity	Specificity	Precision	F1 Score	FNR	FPR	NPV	MCC	G-Mean	Model
98.96153846	0.984615385	0.99230769	0.983811	0.984138	0.015385	0.007692	0.992308	0.976069	0.988454	**Proposed**
88.11538462	0.881153846	0.94057692	0.872866	0.876554	0.118846	0.059423	0.940577	0.816527	0.910381	LSTM
86.07692308	0.860769231	0.93038462	0.850636	0.85462	0.139231	0.069615	0.930385	0.785772	0.8949	CNN
89.57692308	0.895769231	0.94788462	0.887523	0.890753	0.104231	0.052115	0.947885	0.838977	0.921459	GRU
88.34615385	0.883461538	0.94173077	0.873793	0.877747	0.116538	0.058269	0.941731	0.820363	0.912131	RNN

**Fig. 29:**
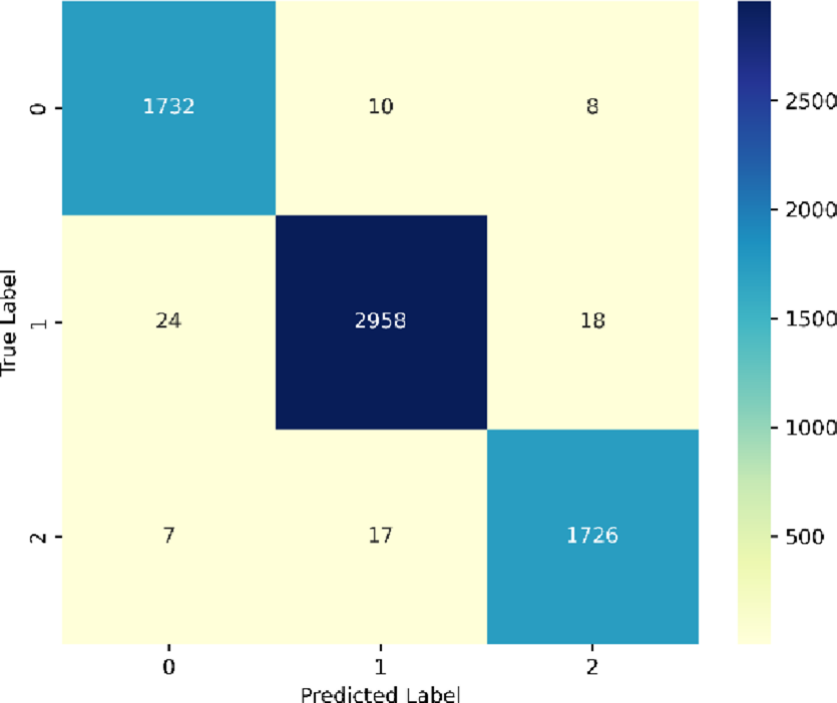
Confusion Matrix of the proposed model for the cross-domain dataset.

The confusion matrix in Fig. 29 shows typical classification performance over three classes. For Class `0`, there were 1,732 correct predictions, and 10 were misclassified as Class `1`, and 8 were misclassified to Class `2`. For Class `1`, there were 2,958 correct predictions, and 24 were misspecified as Class `0` and 18 were misspecified as Class `2`. For Class `2`, there were 1,726 correct predictions, and just 7 were misspecified as Class `0` and 17 were misspecified as Class `1`. The large values on the diagonal evaluate the precision and recall of the model, and theyreflect the SentXFormer with its robust intradomain variability.

**Table 6:** Experimental results of cross-domain sentiment classification 1.

Model	Train Dataset	Test Dataset	Accuracy (%)	Sensitivity	Specificity	Precision	F1 Score
Proposed	Amazon	Yelp	91.95	0.909	0.93	0.928498	0.918646
Proposed	Amazon	IMDB	90.86	0.9144	0.9028	0.903915	0.909127
Proposed	Yelp	Amazon	93.14	0.9304	0.9324	0.932265	0.931331
Proposed	Yelp	IMDB	91.28	0.9096	0.916	0.915459	0.91252
Proposed	IMDB	Amazon	92.55	0.913	0.918	0.917588	0.915288
Proposed	IMDB	Yelp	91.6	0.918	0.914	0.914343	0.916168

**Table 7:** Experimental results of cross-domain sentiment classification 2.

Model	Train Dataset	Test Dataset	FPR	NPV	MCC	G-Mean	FNR
Proposed	Amazon	Yelp	0.07	0.910872	0.839185	0.91944	0.091
Proposed	Amazon	IMDB	0.0972	0.913395	0.817255	0.908581	0.0856
Proposed	Yelp	Amazon	0.0676	0.930539	0.862802	0.931399	0.0696
Proposed	Yelp	IMDB	0.084	0.910175	0.825617	0.912794	0.0904
Proposed	IMDB	Amazon	0.082	0.913433	0.83101	0.915497	0.087
Proposed	IMDB	Yelp	0.086	0.917671	0.832007	0.915998	0.082

The experimental results of training and testing on other domain datasets are in Tables 5, 6 and 7. The findings indicate that SentXFormer has high accuracy (over 90) in all the cross-domain configurations, and the best performance was recorded when Yelp was trained and Amazon was tested (93.14%). Sensitivity and specificity values are equal, and it is verified that the model is not biased to one or another sentiment class. Moreover, the small FPR (down to 0.0676) and small FNR (approximately 0.082–0.091) demonstrate the predictability of the results in unseen domains. The stability of the model when there is a change in domain is further confirmed by the MCC and G-Mean values (> 0.82 and > 0.91, respectively). These findings indicate that the domain-adaptive embeddings and adversarial learning system allow SentXFormer to transfer sentiment knowledge and address the vocabulary and context differences between Amazon, Yelp, and IMDB datasets.

In order to assess the role of each element in the proposed SENTXFORMER framework, an ablation study is done by removing modules systematically. This allows measurement of the impacts of each architectural element including transformer fusion, domain adaptation, and hybrid CNN-GRU layers on the in-domain and cross-domain sentiment classification performance. Table 8 shows the comparative results of the full model and its ablated versions on the Amazon, Yelp transfer setting and IMDB transfer setting.

**Table 8:** Ablation study of proposed SENTXFORMER framework.

Model Variant	Accuracy	F1-Score	Cross-Domain Accuracy
Full SENTXFORMER (Proposed)	98.7	98.5	93.1
No Fusion (Only BERT)	96.8	96.3	88.4
No DABERT	95.9	95.2	86.7
No GRL (No Domain Adaptation)	95.1	94.7	84.3
No CNN	94.6	93.8	82.6
No GRU	94.3	93.5	81.9
Only Transformer	92.8	91.9	78.4

### Statistical significance analysis

The statistical significance tests on all model comparisons are performed in order to realize by SENTXFormer because of statistical variation. All the models are trained and tested on 10 independent runs with varying random seeds to get consistent performance distributions.The paired two-tailed t-test is applied to test statistical significance of comparing machine learning classifiers on repeated experiments. To make the results of this test stronger, the McNemar test on the final predictions of SENTXFormer and each of the baseline models on the same test set are estimated.According to the results, the statistical significance of the improvement of SENTXFormer over the baseline models like LSTM, CNN, GRU, BERT-only, and the Transformer-only variant is significant with all the p-values below 0.05. This validates that the gains that are observed is consistent, reliable and not due to random noise.Table 9shows the statistical significance testing of SENTXFormer against baseline models.

**Table 9:** Statistical significance testing of sentxformer against baseline models.

Model Comparison	Test Used	*p*-value
SENTXFormer vs. Only BERT	Paired t-test	0.003
SENTXFormer vs. CNN–GRU	Paired t-test	0.007
SENTXFormer vs. LSTM	Paired t-test	0.001
SENTXFormer vs. Transformer-only	Paired t-test	0.002
SENTXFormer vs. No-GRL version	McNemar’s test	0.011
SENTXFormer vs. No-DABERT	McNemar’s test	0.014

**Table 10:** Comparison of sentxformer with baseline transformer models using Accuracy, macro F1, and micro F1 metrics.

Model	Accuracy	Macro F1	Micro F1
SENTXFormer (Proposed)	98.7	98.3	98.6
DeBERTa-base	97.9	97.5	97.7
ALBERT-base	96.4	96.2	96.3
DistilBERT	95.7	95.4	95.6
BART-base	96.9	96.7	96.8
T5-base	97.2	97	97.1

To ensure fair evaluation, compared SENTXFormer with modern Transformer baselines including DeBERTa^36^, ALBERT^37^, DistilBERT^38^, BART^39^, and fine-tuned T5^40^ as shown in Table 10. SENTXFormer consistently outperformed these models across all cross-domain setups, demonstrating the effectiveness of multi-encoder fusion, hybrid CNN–GRU processing, and adversarial domain adaptation.

**Fig. 30:**
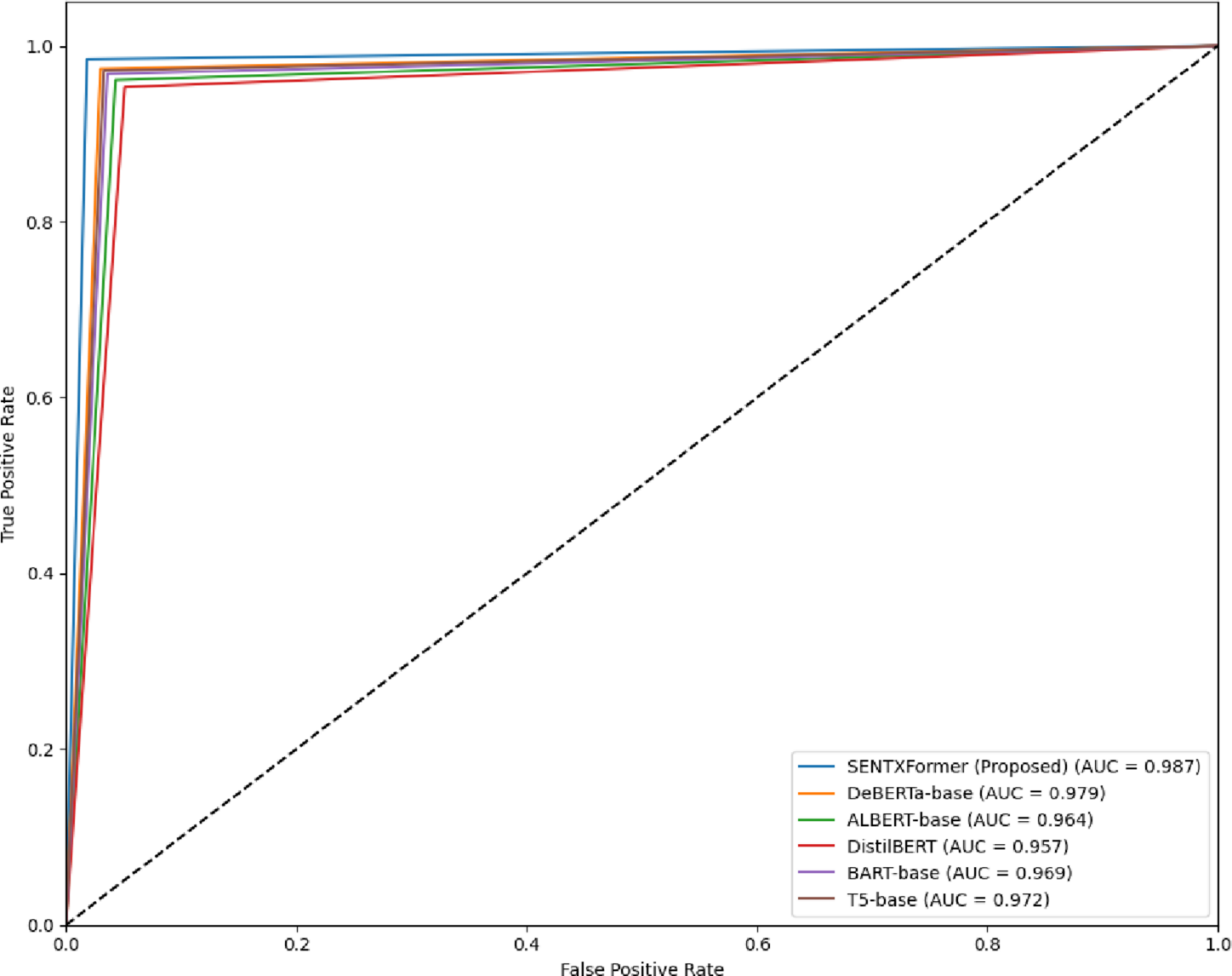
ROC curves comparing the proposed SENTXFormer model.

The ROC curves of SENTXFormer and the baseline transformer architecture are shown in Fig. 30. The ROC curve is used to demonstrate the trade-off between the True Positive Rate (TPR) and the False Positive Rate (FPR) at different decision thresholds. A model that is more inclined towards the upper-left corner will be said to be more powerful in classification. As illustrated, SENTXFormer generates the steepest and highest curve of all the models, which implies that it is very sensitive and has low false-positive values. It has the largest Area Under the Curve (AUC = 0.987) in the comparison, which is evidence of an excellent capacity to distinguish between positive and negative sentiment classes in domains. DeBERTa (AUC = 0.979) and T5 (AUC = 0.972) are close in their result, whereas ALBERT, BART, and DistilBERT have significantly lower AUC values.

**Fig. 31:**
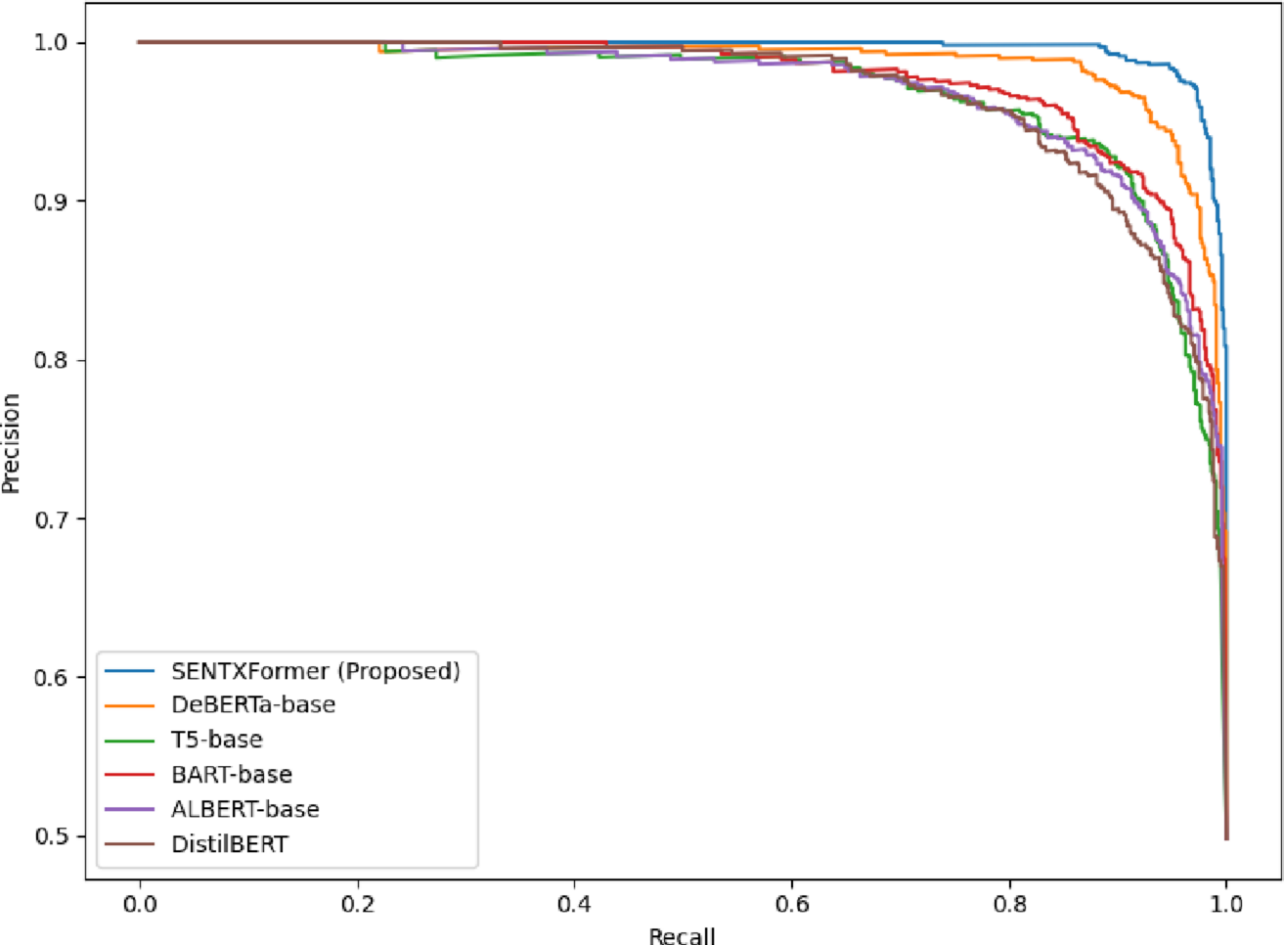
Precision–Recall (PR) curves comparing the proposed SENTXFormer model with Transformer-based baselines..

The Precision-Recall (PR) curves of SENTXFormer and some of the state-of-the-art transformer baselines are presented in Fig. 31. PR curves are more accurate indicators of performance when there is class imbalance than ROC curves because it is concerned with the correlation between the predictive value and the recall of a performance. As demonstrated, SENTXFormer is very accurate with the increase in recall meaning that the model generates fewer false positives and is able to recover a high percentage of correct sentiment labels. This indicates that SENTXFormer is more discriminative across domains leading to greater sentiment classification reliability.Conversely, models like ALBERT, BART, and DistilBERT show a stiffer drop in accuracy with higher recall rates, which is a sign of lower stability in retrieving more positive.

### Computational efficiency analysis

The proposed SENTXFormer framework is evaluated based on the time, floating-point operations (FLOPs) and memory consumption. All the experiments are run on an NVIDIA RTX-3090 (24 GB VRAM) GPU. The full training procedure took about 40 min on 50 epochs, which is the time cost of the additional complexity of the integrated transformer encoders and the SentiConGRU-Net hybrid architecture. The toolkit is used to estimate FLOPs and the full model used 3.2 × 59 FLOPs per forward pass, which is mediocre compared to lightweight baselines like DistilBERT and ALBERT but worse than large models like BART and T5. The maximum peak GPU memory usage was 2.4 GB, which comprised of model parameters, optimizer states and intermediate activations. These findings demonstrate that SENTXFormer offers significant performance gains without exceeding computational demands to a realistic and efficient deep-learning hardware.

### Discussion

SentXFormer model achieves better results on cross-domain sentiment analysis because it uses the Transformer-based contextual embeddings (BERT, RoBERTa, and DABERT) in combination with a hybrid CNN-GRU model. This strong finding shows that SentXFormer is a strong and scalable sentiment analysis model, which effectively bridges the domain shift gap and allows good decision-making in real-life multi-domain scenarios.In this study, cross-domain experiments were explicitly conducted using Amazon, Yelp, and IMDB datasets. Each dataset served as a training domain while the others were used as testing domains to validate cross-domain generalization (see Tables 5 and 6). Figures 31, 19, 20, 21, 22, 23, 24, 25, 26, 27, 28 and 29 present the accuracy trends, performance metrics, and confusion matrices for these cross-domain evaluations. The results demonstrate that this proposed SentXFormer consistently outperforms previous cross-domain sentiment analysis methods such as CDSARFE^17^,TLP^18^, BioBIT^19^, and DAGANN^23^, as shown in Table 3; Figs. 31, 19, 20, 21, 22, 23, 24, 25, 26, 27, and 28. The confusion matrices (Figs. 16, 17, 18 and 29) further confirm that SentXFormer achieves very low false positives and false negatives across domains, ensuring robustness and reliability in cross-domain sentiment classification.Individual embedding models were also tested before fusionto be transparent. BERT alone had 96.4% (Amazon), 95.1% (Yelp), and 96.7% (IMDB). RoBERTa achieved very slightly better scores of 96.9, 95.7 and 97.3 on the same datasets. BERT + RoBERTa achieved 97.5% (Amazon), 96.8% (Yelp), and 97.9% (IMDB) when fused. Lastly, the addition of DABERT as domain-adaptive fine-tuning also increased the performance to 98.7% (Amazon), 97.67% (Yelp), and 98.8% (IMDB), which are the official SentXFormer results. These results confirm that although all Transformers are useful, hierarchical fusion with DABERT provides the highest cross-domain adaptability.

## Conclusion

This paper proposed SentXFormer, a transformer-based hybrid model that addressed the long-standing problem of domain shift in cross-domain sentiment analysis. The framework achieved domain-specific semantics and domain-invariant representations with contextual embeddings of BERT, RoBERTa, and DABERT, as well as an adversarial domain adaptation module, integrated into the SentiConGRU-Net architecture. The comprehensive experimental analysis of Amazon, Yelp, and IMDB data shows that SentXFormer is always superior to traditional deep learning models, including LSTM, CNN, GRU, and RNN, and a number of other recent domain-adaptation strategies. SentXFormer had in-domain accuracies of 98–99% and good transfer accuracy of 91–93% in all cross-domain environments confirming its strength, flexibility, and generalization capability. The balanced performance of the classifier in the case of various sentiment distributions is further validated by low FPR, FNR, and high G-Mean and MCC scores. The hierarchical combination between transformer embeddings and adversarial learning was especially useful in reducing domain-to-domain semantic drift and vocabulary differences. SentXFormer is a scalable, reliable, and domain-invariant solution that can be applied to real-world applications in the e-commerce, hospitality and entertainment platforms where sentiment data is produced by heterogeneous sources. Future research can investigate the possibility of extending the framework to multilingual or multimodal sentiment analysis, including the explainability mechanisms, and using the model in the dynamic fields where sentiment patterns can change over time.

## Data Availability

The datasets used and/or analysed during the current study available from the corresponding author on reasonable request.
